# Catalytically distinct metabolic enzyme isocitrate dehydrogenase 1 mutants tune phenotype severity in tumor models

**DOI:** 10.1016/j.jbc.2025.108477

**Published:** 2025-04-04

**Authors:** Ashley V. Schwartz, Grace Chao, Mikella Robinson, Brittany M. Conley, Mowaffaq Adam Ahmed Adam, Grace A. Wells, An Hoang, Elene Albekioni, Cecilia Gallo, Joi Weeks, Katelyn Yunker, Giovanni Quichocho, Uduak Z. George, Ingrid Niesman, Carrie D. House, Şevin Turcan, Christal D. Sohl

**Affiliations:** 1 Computational Science Research Center, San Diego State University, San Diego, California, USA; 2 Department of Biology, San Diego State University, San Diego, California, USA; 3 Department of Chemistry and Biochemistry, San Diego State University, San Diego, California, USA; 4 Department of Mathematics and Statistics, San Diego State University, San Diego, California, USA; 5 Electron Microscope Facility, San Diego State University, San Diego, California, USA; 6 Neurology Clinic and National Center for Tumor Diseases, Heidelberg University Hospital and Heidelberg University, Heidelberg, Germany

**Keywords:** epigenetics, dehydrogenase, enzyme mutation, oncogene, transcriptomics

## Abstract

Mutations in isocitrate dehydrogenase 1 (IDH1) impart a neomorphic reaction that produces D-2-hydroxyglutarate (D2HG), which can inhibit DNA demethylases to drive tumorigenesis. Mutations affect residue R132 and display distinct catalytic profiles for D2HG production. We show that catalytic efficiency of D2HG production is greater in IDH1 R132Q than R132H mutants, and expression of IDH1 R132Q in cellular and xenograft models leads to higher D2HG concentrations in cells, tumors, and sera compared to R132H. Though expression of IDH1 R132Q leads to hypermethylation in DNA damage pathways, DNA hypomethylation is more notable when compared to IDH1 R132H expression. Transcriptome analysis shows increased expression of many pro-tumor pathways upon expression of IDH1 R132Q *versus* R132H, including transcripts of EGFR and PI3K signaling pathways. Thus, IDH1 mutants appear to modulate D2HG levels *via* altered catalysis and are associated with distinct epigenetic and transcriptomic consequences, with higher D2HG levels appearing to be associated with more aggressive tumors.

Isocitrate dehydrogenase 1 (IDH1) is a homodimeric enzyme that catalyzes the reversible NADP^+^-dependent oxidation of isocitrate (ICT) to α-ketoglutarate (αKG) in the cytoplasm and peroxisomes. Somatic mutations in IDH1 have been implicated in lower grade gliomas, chondrosarcomas, and intrahepatic cholangiocarcinomas (1). These mutations typically ablate this conventional activity, but more importantly drive a neomorphic function: the NADPH-dependent conversion of αKG to the oncometabolite, D-2-hydroxyglutarate (D2HG) (2). D2HG can competitively inhibit αKG-dependent enzymes such as the 5-methylcytosine hydroxylase TET2 and JmjC lysine demethylases to result in increased DNA and histone methylation, respectively (3, 4, 5, 6, 7, 8). We showed previously that mutations in IDH1 cause the CpG island methylator phenotype (CIMP) (9), a clinical diagnostic and prognostic feature of tumors (10).

Tumor-driving IDH1 mutations affect residue R132, with R132H and R132C the most common, though more rare mutations, including R132G, R132L, R132S, and R132Q have been reported in patients (2, 11, 12, 13). Residue 132 interacts with the C3 carboxylate of ICT (2, 14, 15, 16) and stabilizes an important regulatory domain, the α10 helix (2, 14, 16, 17, 18). Loss of arginine at residue 132 helps favor a closed conformation to drive the equilibrium towards αKG and NADPH binding, explaining at least in part why there is some permissiveness in mutations seen at this residue in patients (2, 16). There has been interest in determining if different IDH1 mutations lead to different features in IDH1-driven tumors. Glioma tissue (expressing IDH1 R132H/C/G mutations) and cell lines (expressing IDH1 R132H/C/G/S/L mutations) showed varying concentrations of D2HG depending on the IDH1 mutation present, with R132H associated with the lowest levels of D2HG (19, 20). We previously reported that purified IDH1 R132Q was far more catalytically efficient at D2HG production than R132H (21, 22) due to activating structural features (23). An extensive study on astrocytomas that were either R132H-mutated or had non-canonical IDH1/2 mutations showed that non-R132H IDH1/2-mutated tumors had overall increased DNA methylation, decreased gene expression, and better prognosis, suggesting an intriguing possibility of differences in phenotype intensity within IDH1 mutants and/or among IDH1 and IDH2 mutants (24), while others have shown that there is no difference in overall survival when comparing non-canonical and canonical IDH1 mutations found in grade 4 astrocytomas (25). Other work has indicated that adverse progression-free survival in patients with lower grade mutant IDH-driven gliomas can be predicted by increasing concentrations of 2HG and glutamate (26). Together, these studies highlight an important unresolved question: do different concentrations of D2HG resulting from different IDH1 mutations lead to changes in tumor phenotype or phenotype intensity? If increased catalytic rates conferred among some IDH1 mutants can indeed lead to higher D2HG concentrations in tumor models, understanding the impact of these mutations on tumor growth rates, epigenetic, and transcriptomic profiles can ultimately help us better predict patient prognosis.

Here, we sought to determine the *in vitro* and *in vivo* consequences of two IDH1 mutants with profoundly different kinetic profiles. We generated ectopic mouse tumor xenografts from either glioma or sarcoma cell lines that we modified to stably over-express IDH1 WT, R132H, or R132Q, and performed comprehensive epigenomic and transcriptomic analyses on these tumors. We found that tumors expressing IDH1 R132Q had increased D2HG levels and larger tumors compared to WT and R132H, but the glioma and chondrosarcoma xenograft tumor models had highly variable consequences on genome methylation and gene expression, with increased pro-tumorigenic pathways upregulated in xenograft tumors upon expression of IDH1 R132Q.

## Results

### D2HG levels in IDH1 R132Q tumor models are higher

Though we have shown that IDH1 R132Q mutant homodimers produce D2HG more efficiently than R132H mutant homodimers (21, 22, 23) here we made 1:1 mixtures of purified WT and mutant IDH1 enzymes to mimic the heterozygosity found in patients. These IDH1 WT:R132Q mixtures, which may form heterodimers, retained superior catalytic efficiency compared to WT:R132H, with 9.3-fold more efficient D2HG production (Fig. S1). This was driven both by an increase in *k*_cat_ and decrease in *K*_m_.

**Figure 1:**
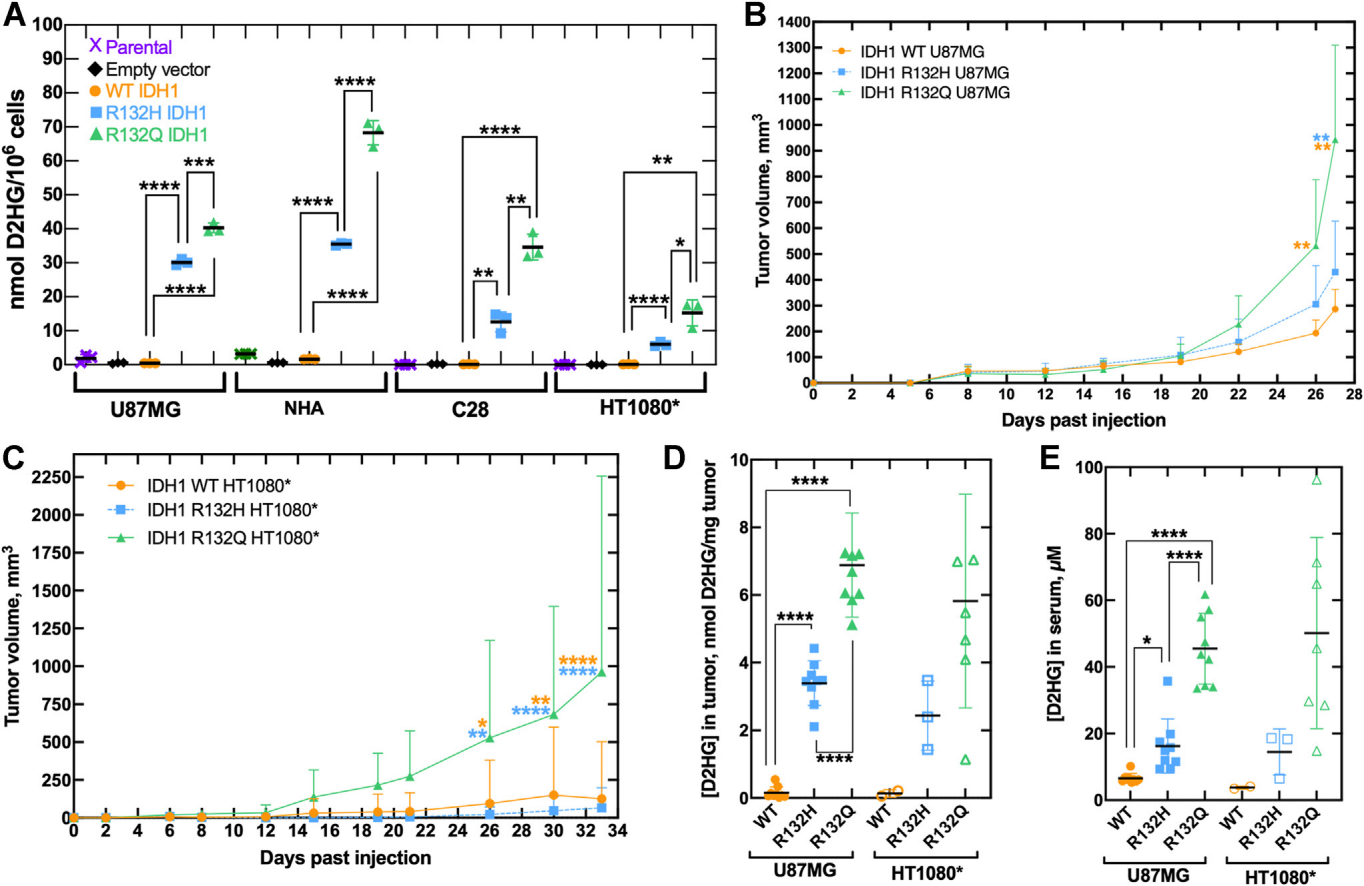
**Catalytic efficiency of IDH1 mutants drive D2HG levels in biochemical, cellular, and *in vivo* models.***A*, parental (*purple* x’s), empty vector (EV, *black diamonds*), HA-tagged IDH1 WT (*orange circles*), HA-tagged IDH1 R132H (*blue squares*), or HA-tagged IDH1 R132Q (*green triangles*) was stably overexpressed in glioma cells (U87MG), normal human astrocytes (NHA), chondrocytes (C28), and fibrosarcoma cells where an endogenous IDH1 R132C mutation was removed (*i.e.* HT1080^+/−^, denoted HT1080∗). Cellular D2HG levels were quantified in three biological replicates per cell type. Significant differences involving parental and empty vector (EV) are not highlighted to improve clarity. *B*, U87MG and (*C*) HT1080∗ lines stably overexpressing IDH1 WT, R132H, or R132Q were used to generate mouse xenografts and tumor volume was measured over time. All 27 mice (nine per sample group) formed U87MG xenograft tumors, and the mean of the nine biological replicates with S.D. shown in (*B*). Only two, three, or seven mice formed HT1080∗ xenograft tumors upon IDH1 WT, R132H, or R132Q expression, respectively, and the mean of these biological replicates with S.D. is shown in (*C*). In (*B* and *C*), significant differences from IDH1 R132Q are indicated with *p* value stars colored according to the corresponding comparison group (WT in *orange*, R132H in *blue*). *D*, tumors and (*E*) sera were harvested from the mice described in (*B* and *C*), and D2HG was quantified. In panels (*A*–*E*), *p* values were determined by unpaired t tests (*A*), two-way repeated measures ANOVA, Tukey *post hoc* test (*B* and *C*), or ordinary one-way ANOVA (*D* and *E*), with ∗∗∗∗*p* ≤ 0.0001, ∗∗∗*p* ≤ 0.001, ∗∗*p* ≤ 0.01, ∗*p* ≤ 0.05 for pairwise comparisons between IDH1 WT, R132H, and R132Q.

Though catalytic parameters identify important intrinsic enzyme properties, this ignores the complexities of the cellular environment. As patient-derived IDH1 R132Q-driven tumor cells were unavailable due to the rarity of this mutation, we generated isogenic cell lines stably overexpressing IDH1 WT, R132H, R132Q, or empty vector (EV). We selected well-characterized cancer-derived and non-cancer-derived cell lines commonly used to model IDH1 mutations in gliomas and chondrosarcomas: normal human astrocytes (NHA), U87MG glioma cells, C28 chondrocytes, and a modified HT1080 sarcoma line with the endogenous IDH1 R132C mutation removed to generate IDH1^−/+^, designated here as HT1080∗. Though the HT1080 cell line had originally been characterized as a fibrosarcoma, discovery of the endogenous IDH1 R132C mutation has led to speculation that it is more likely a de-differentiated chondrosarcoma, and thus here we will refer to this cell line as a chondrosarcoma model, rather than a fibrosarcoma model (27, 28). Interestingly, we were unable to generate clones that sustained high expression of IDH1 R132Q (Fig. S2). Despite consistently lower expression of IDH1 R132Q relative to R132H, we nonetheless found that D2HG concentrations were significantly higher in this more catalytically active mutant (Fig. 1*A*) in both cancerous (U87MG, HT1080∗) and non-cancerous cell lines (NHA, C28).

We next sought to understand the consequences of mutants with differing catalytic efficiency in *in vivo* models of mutant IDH1 tumors. To increase the likelihood of tumor formation, we used IDH1 WT-, R132H-, or R132Q-expressing glioma (U87MG) and sarcoma (HT1080∗) cells to generate subcutaneous mouse xenografts. For the U87MG series, tumors formed in 100% of the mice (27/27, nine per group), with no significant differences in days to tumor initiation or tumor density (Fig. S3). However, U87MG tumors expressing IDH1 R132Q had significantly higher volume (Fig. 1*B*), mass, and growth rate (Fig. S3) compared to IDH1 WT and R132H. Tumors were formed in 22% (2/9), 30% (3/10), and 78% (7/9) of the IDH1 WT-, R132H-, or R132Q-expressing HT1080∗ mouse xenografts, respectively. Here, tumor volume was larger (Fig. 1*C*), and tumor initiation occurred significantly earlier for IDH1 R132Q mutants compared to WT and R132H, though tumor mass and tumor density showed no significant difference (Fig. S4).

We harvested all available tumors and sera from mice and found that D2HG levels were significantly higher in IDH1 R132Q-expressing U87MG xenografts relative to WT and R132H (Fig. 1, *D* and *E*), even though protein expression of IDH1 R132H and WT was higher compared to R132Q (Fig. S5 and Table S1). These trends were recapitulated in the HT1080∗ xenografts, though significance was not reached (Fig. 1, *D* and *E* and Table S1). There were few obvious trends among D2HG concentrations and tumor features within biological replicates, though modest correlation in serum D2HG concentration and tumor mass as a percentage of body mass (Fig. S6) was observed. Together, these tumor models showed that expression of IDH1 R132Q was associated with more catalytically efficient D2HG production, higher cellular, tumor, and serum levels of D2HG, and larger tumors compared to R132H.

### Epigenomic and transcriptomic features of mutant IDH1 chondrosarcoma models

**Figure 2:**
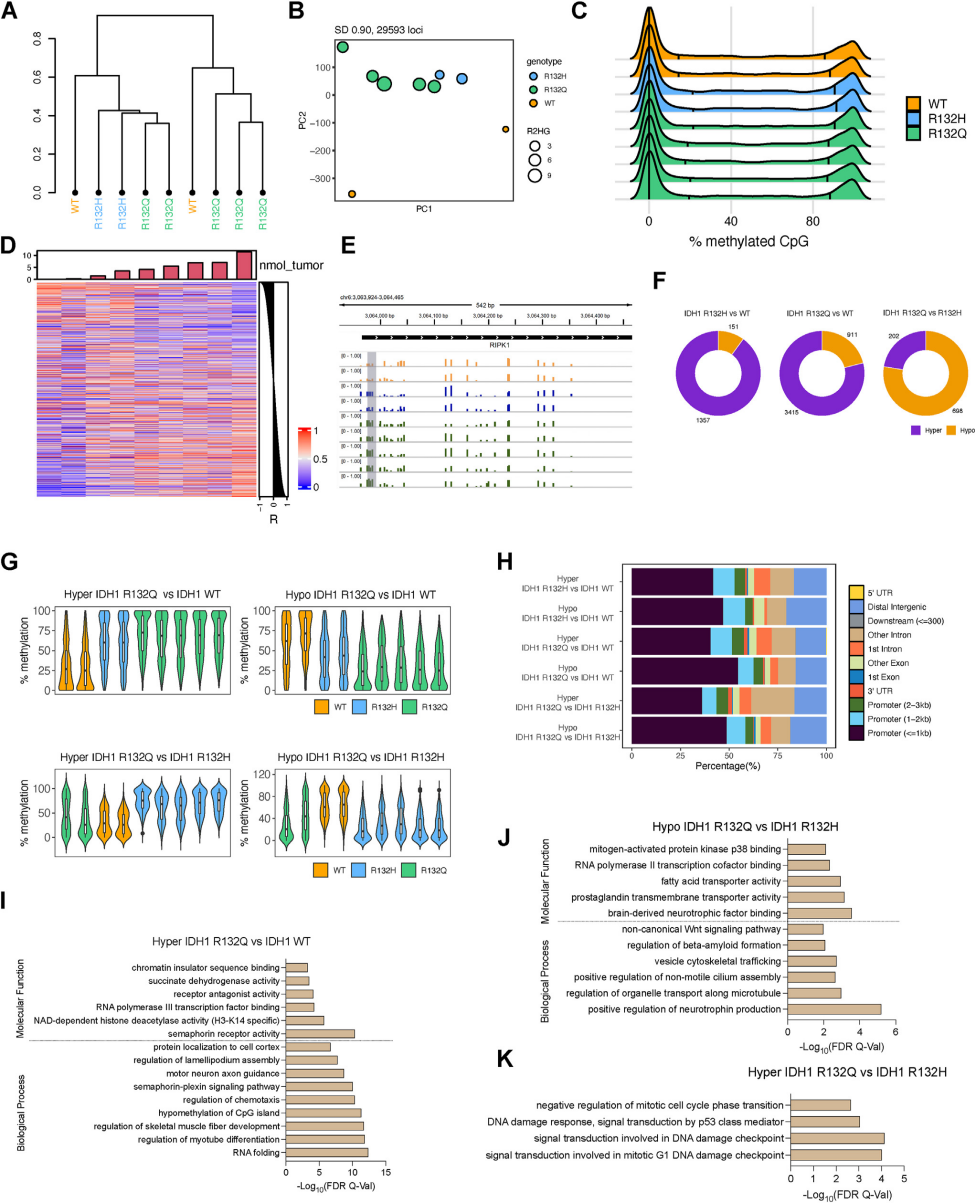
**IDH1 R132Q-induced methylation remodeling differs from IDH1 R132H in HT1080∗ tumors.** Two of two, three of three, and five of seven (randomized selection) of the xenograft tumors that formed HT1080∗ xenograft tumors upon IDH1 WT, R132H, or R132Q expression, respectively, were evaluated as biological replicates by RRBS, though one of the R132H xenograft tumors failed analysis. *A*, hierarchical clustering of the most variable CpG sites (S. D. > 0.90, n = 29,593) in the HT1080∗ RRBS cohort. *B*, PCA plot of the most variable CpG sites (S. D. > 0.90) in the HT1080∗ RRBS cohort. The size of each dot corresponds to the level of D2HG (nmol tumor) measured in each tumor sample. Colors indicate genotype. *C*, global methylation profiles of the HT1080∗ RRBS tumor cohort showing percent methylation values of CpG sites for each sample. *Black vertical* lines indicate quantiles; colors indicate genotype. *D*, heatmap showing the correlation between D2HG (nmol_tumor) and methylation values of the top 1% of the most variable CpG sites. Pearson correlation coefficients (R) are shown on the *right* of the heatmap. The amount of D2HG in tumors is depicted as bar plot above the heatmap. *E*, number of differentially methylated CpG sites (DMS, % methylation difference ≥ 20 and q-value < 0.05) are shown. *Left*, IDH1 R132H *versus* IDH1 WT; *middle*, IDH1 R132Q *versus* IDH1 WT; *right*, IDH1 R132Q *versus* IDH1 R132H. *F*, distribution of the genomic features for the hyper- and hypo-methylated loci for all comparisons. *G*, violin plots showing the distribution of percent (%) methylation of hypermethylated loci in IDH1 R132Q *versus* IDH1 WT (*top left*), hypomethylated loci in IDH1 R132Q *versus* IDH1 WT (*top right*), hypermethylated loci in IDH1 R132Q *versus* IDH1 R132Q (*bottom left*), and hypomethylated loci in IDH1 R132Q *versus* IDH1 R132H (*bottom right*). For clarity, samples not included in the pairwise statistical comparisons are also plotted. *H*, Distribution of the genomic features for the hyper- and hypo-methylated loci for all comparisons. *I*–*K*, gene ontology enrichment (using GREAT toolbox) of the (*I*) hypermethylated (hyper) loci in IDH1 R132Q compared to IDH1 WT (*J*), hypomethylated (hypo) loci in IDH1 R132Q *versus* IDH1 R132H, and (*K*) hypermethylated loci in IDH1 R132Q *versus* IDH1 R132H samples. hyper, hypermethylated; hypo, hypomethylated.

To probe the mechanism(s) behind the differences in tumor size in the IDH1 R132Q *versus* R132H and WT tumors, we used reduced representation bisulfite sequencing (RRBS) to analyze genome-wide DNA methylation in representative xenograft tumors. We first performed clustering analysis for the HT1080∗ xenografts based on the degree of CpG methylation of the most variable probes with the highest SD (Fig. 2*A*). There was modest clustering based on genotype, with HT1080∗ xenografts expressing IDH1 WT having the most variation. This is perhaps unsurprising given the wide span in tumor weight and low *n* for WT IDH1-expressing HT1080∗ tumors (Table S1). Better separation of the two mutants was achieved *via* PCA that included D2HG tumor concentrations (Fig. 2*B*). When comparing distribution of methylation values for all CpG sites, there was a modest shift in hypermethylation in mutant *versus* WT HT1080∗ xenografts (Fig. 2*C*). We then analyzed the methylation levels of CpG sites that correlated with tumor D2HG amounts in HT1080∗ xenografts and identified the loci that were positively correlated with D2HG (Fig. 2*D*). Next, we identified the pairwise differentially methylated loci among all three genotypes in HT1080∗ tumors and found IDH1 R132Q induced hypermethylation in a larger number of CpG sites than R132H when compared to WT (Fig. 2*E*–*G*). In general, both IDH1 R132H and R132Q chondrosarcoma tumor models showed the same hypermethylation localization trends compared to WT, with promoter and intronic regions showing the most hypermethylation (Fig. 2*H*). However, when comparing the two mutants, significantly increased hypomethylation was observed upon R132Q expression *versus* R132H (Figs. 2, *F*–*G* and S7), especially in promoter regions and CpG islands (Figs. 2*H* and S8).

To compare the expression patterns of genes in the WT-, R132H-, and R132Q-driven xenografts, we also performed RNA-seq analysis. Consistent with both mutants being hypermethylated within the promoter regions relative to WT, which would predict increased gene silencing, there were more down-regulated than up-regulated genes for IDH1 R132H and R132Q HT1080∗ xenografts relative to WT (Table S2). Similarly, consistent with an increase in hypomethylation of CpG sites in R132Q *versus* R123H, which would predict an increase in gene expression, there were more upregulated genes in R132Q than downregulated compared to R132H (Table S2). Together, these data suggest that in chondrosarcoma tumor models, expression of IDH1 R132 mutants is associated with increased hypermethylation compared to WT, while IDH1 R132Q expression is associated with increased regions of hypomethylation compared to R132H.

### Pathways hypermethylated and downregulated in IDH1 R132Q chondrosarcoma models

Hypermethylated pathways in IDH1 R132Q-*versus* R132H-expressing chondrosarcoma models included cell cycle, DNA damage response, and DNA damage checkpoint signal transduction (Fig. 2*H*), suggesting that the DNA damage response may be inhibited. Notably, IDH1 R132Q had higher methylation at the promoter of mitotic arrest deficient 2-like protein 2 (*MAD2L2*, also known as *REV7*) relative to R132H. Consistent with this increased methylation, RNAseq analysis showed expression of *MAD2L2* trended lower in R132Q-expressing HT1080∗ tumors compared to R132H (*p* = 0.028, *p*_adj_ = 0.21). MAD2L2 plays a role in translesion DNA synthesis and double stranded DNA break repair, promoting progression of stalled replication forks past lesions and driving DNA repair pathways (29, 30). Loss of MAD2L2 can lead to genomic damage as a result of stalled replication forks (31) and increased proliferation and migration (32), and thus has been posited to be a tumor suppressor though it may have an oncogenic role in some contexts (33). Significant hypermethylation within the promoter of growth arrest and DNA-damage-inducible protein γ (*GADD45G*) occurred in IDH1 R132Q *versus* R132H HT1080∗ xenograft tumors, though this gene did not appear in our RNAseq analysis. GADD45G is a tumor suppressor that is involved in a host of cellular processes, including DNA repair (34), cell cycle arrest (35), and apoptosis (36), and its silencing or downregulation has been implicated in AML (37) and breast cancer (38). GADD45G promotes cell differentiation through negative regulation of the phosphoinositide 3-kinase/AKT/mTOR (PI3K/AKT/mTOR) pathway, and, consequently, its downregulation leads to activation of this pathway to drive cancer (39).

Many pathways showed significant hypermethylation in IDH1 R132Q vs. WT HT1080∗ xenografts, including pathways involved in altered transcription and cell movement (Fig. 2*I*). Pathways associated with morphogenesis and differentiation were also hypermethylated in R132Q relative to WT, including significant hypermethylation of Wnt pathway members *WNT9A*, *WNT10A*, frizzled-9 (*FZD9*), palmitoleoyl-protein carboxylesterase (*NOTUM*), T-cell specific transcription factor 7 (*TCF7*), and naked cuticle 1 (*NKD1)*. Indeed, RNAseq analysis showed that *WNT9A* was significantly down-regulated in IDH1 R132Q *versus* WT HT1080∗ xenografts, with *FZD9* and *TCF7* transcripts trending down but not achieving significance when adjusted for multiple comparisons (Table S3).

**Figure 3:**
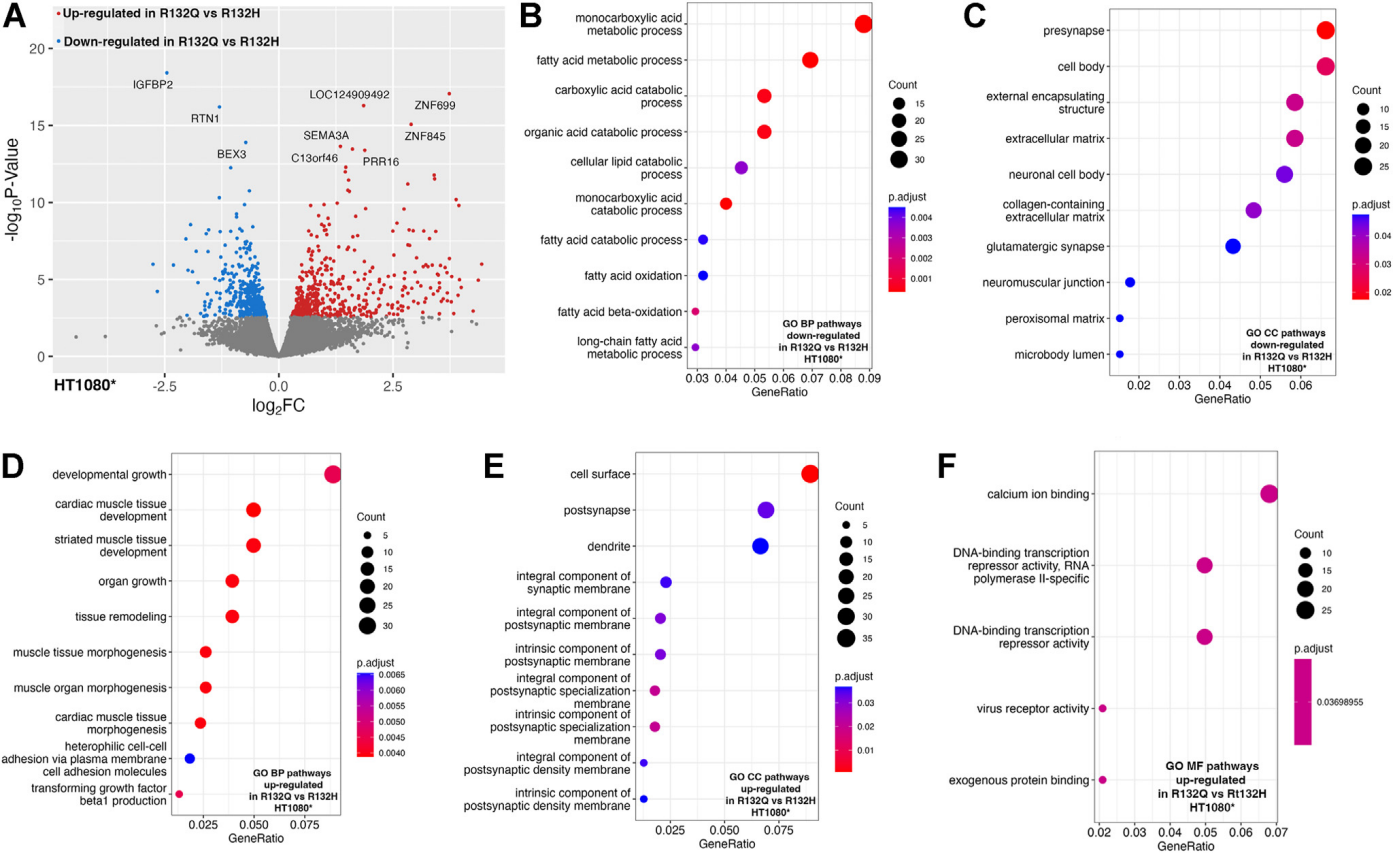
**Transcriptome analysis using RNAseq of HT1080∗ xenograft tumors comparing IDH1 R132Q and IDH1 R132H.** Two of two, three of three, and five of seven (randomized selection) of the xenograft tumors that formed HT1080∗ xenograft tumors upon IDH1 WT, R132H, or R132Q expression, respectively, were evaluated as biological replicates by RNAseq. Downregulated pathways refer to categories where most genes show decreased expression levels in R132Q *versus* R132H IDH1. Upregulated pathways refer to categories where most genes show increased expression levels in R132Q *versus* R132H IDH1. Number of genes is indicated in the count, with the *p*_adjusted_ value indicated by color. *A*, volcano plot of differentially expressed transcripts comparing expression of IDH1 R132Q *versus* IDH1 R132H. *B*, biological pathways (BP) downregulated in IDH1 R132Q *versus* IDH1 R132H. *C*, cellular component (CC) pathways downregulated in IDH1 R132Q *versus* IDH1 R132H. *D*, BP upregulated in IDH1 R132Q *versus* IDH1 R132H. *E*, CC pathways upregulated in IDH1 R132Q *versus* IDH1 R132H. *F*, molecular function (MF) pathways upregulated in IDH1 R132Q *versus* IDH1 R132H.

RNAseq analysis also identified transcript downregulation not necessarily driven by genome hypermethylation. For example, transcripts involved in collagen/extracellular matrix (ECM) pathways were downregulated upon IDH1 R132Q expression compared to both R132H and WT HT1080∗ xenografts (Figs. 3 and S9). This included decreased *COL9A2* transcripts upon expression of IDH1 R132Q *versus* WT, a type IX collagen often downregulated in high-grade *versus* low-grade chondrosarcomas (40) (Table S3). Annexin-A6 (*ANXA6*) transcripts were also downregulated in R132Q-expressing HT1080∗ xenografts compared to both R132H and WT chondrosarcoma models (Table S3). ANXA6 mediates cholesterol transport and participates in endocytosis and exocytosis (41) and can serve as a tumor suppressor due to its ability to negatively regulate EGFR phosphorylation (42). Supportive of removing a negative regulator, *EGFR* expression was higher in R132Q *versus* R132H and WT HT1080∗ xenografts (Table S3).

Lipid catabolism and lipid oxidation pathways were also downregulated upon expression of IDH1 R132Q *versus* R132H (Fig. 3), suggesting that the use of lipids as a fuel source is more critical in R132H-expressing xenografts than R132Q; indeed, β-oxidation has been shown to be an important adaptive mechanism in R132H tumor models (43). Changes in metabolite levels can help support evidence of altered lipid metabolism; for example, accumulation of dihydroxyacetone phosphate (DHAP) and glycerol-3-phosphate (G3P) can indicate evidence of lipid biosynthesis, while decreased levels are associated with lipid oxidation (44, 45). Metabolomic analysis of xenograft tissue supported a possible role of lipid oxidation being downregulated in IDH1 R132Q *versus* R132H in our chondrosarcoma models. Elevated DHAP and G3P levels, associated with lipid biosynthesis as opposed to lipid oxidation, were observed in IDH1 R132Q HT1080 xenografts and cells compared to R132H, but this trend was not seen in U87MG xenograft and cellular samples (Fig. S10, *A*–*D*).

Our RNA-seq analysis also indicated that monocarboxylic, carboxylic, and organic acid catabolism was downregulated in IDH1 R132Q *versus* R132H-expressing HT1080∗ xenografts (Fig. 3). These catabolic pathways can include breakdown of the branched chain amino acids (BCAAs) leucine, isoleucine, and valine. Decreased catabolism of these amino acids would presumably result in their buildup, and indeed, metabolomic analysis of HT1080∗ xenografts showed increased BCAAs levels in IDH1 R132Q-expressing cells and tumors compared to R132H (Fig. S10, *E* and *F*). BCAAs were also increased, though to a lesser degree, when comparing IDH1 R132Q *versus* R132H expression in U87MG tumors (Fig. S10, *G* and *H*), though RNA-seq did not indicate these pathways were significantly downregulated in U87MG xenografts when comparing the two mutants. BCAAs also play a role in lipid metabolism; for example, adipogenesis regulates levels of BCAAs by directing the upregulation of BCAA catabolism as these amino acids can serve as precursors for fatty acid and sterol biosynthesis (46). Overall, compared to IDH1 R132H, R132Q-expressing chondrosarcoma tumor models experienced significant changes in pathways including DNA damage, Wnt and EGFR signaling, lipid metabolism, carboxylic acid catabolism, and collagen/ECM remodeling likely facilitated by altered CpG methylation and as well as by non-CpG-methylation mechanisms.

### Pathways hypomethylated and upregulated in IDH1 R132Q chondrosarcoma models

As hypomethylated CpG regions were a prominent feature of IDH1 R132Q-expressing HT1080∗ xenografts compared to R132H, we also assessed the pathways affected by decreased methylation, and thus likely upregulated. Pathways predicted to be hypomethylated in IDH1 R132Q *versus* R132H chondrosarcoma models involved p38 MAPK, which helps regulate cell differentiation, growth, and death (47), RNA pol II transcription cofactor binding, vesicle trafficking, fatty acid and organelle transport pathways, and non-canonical Wnt signaling that can drive proliferation and migration in cancer (48) (Fig. 2*J*). Thus, Wnt signaling alterations resulted from both hypermethylation and hypomethylation in IDH1 R132Q-expressing tumors.

Similarly, GO pathways upregulated based on increased transcripts in IDH1 R132Q *versus* R132H HT1080∗ xenografts included pathways associated with growth, remodeling, morphogenesis, and transcription repression (Fig. 3), but there was minimal overlap of genes significantly hypomethylated with elevated transcripts. For example, the *MRAS* promoter was hypomethylated in IDH1 R132Q-expressing xenografts compared to R132H, though this did not translate to a significant increase in *MRAS* expression. To understand the consequences of a less methylated genome in IDH1 R132Q than R132H, we also compared pathways with hypomethylation and increased transcripts in R132Q *versus* WT. We observed hypomethylation of pathways including vesicle loading of neurotransmitters, and indeed RNAseq analysis highlighted an increase in pathways associated with endocytic vesicles, and neuronal projection and axon development/guidance (Fig. S9).

RNAseq analysis again showed many cancer pathways upregulated in IDH1 R132Q-expressing HT1080∗ xenografts *versus* R132H outside of genes identified in RRBS analysis, including proto-oncogenes c-myc (*MYC*), *EGFR*, vascular endothelial growth factor c (*VEGFC*), Ras-like proto-oncogene A (*RALA*), casitas B-lineage lymphoma E3 ubiquitin ligase (*CBL*), and ETS proto-oncogene 1 (*ETS1*) (Figs. 3, S9, and S11). Importantly, activation of c-myc *via* the Wnt pathway can stimulate the cell cycle in part by activating expression or stimulating the accumulation of cyclin dependent kinase 6 (CDK6) and cell-cycle regular cyclin D2 (CCND2) in early G1 phase to promote transition to S phase to support proliferation (49, 50, 51). *MYC*, *CDK6*, and *CCND2* transcripts were all significantly higher in IDH1 R132Q-expressing HT1080∗ xenografts *versus* R132H (Table S3). Though changes in *TP53* expression did not reach significance, tumor protein p53 inducible protein 3 (*TP53I3*) transcripts, which is induced by p53 and serves as a marker for pro-apoptosis (52), were significantly lower in IDH1 R132Q HT1080∗ xenografts than R132H (Table S3). Indeed, transcripts of caspase-9 (*CAS9*), which drives apoptosis (53), were marginally significantly lowered upon R132Q expression *versus* R132H with a *p* < 0.05 before correcting for multiple comparisons (Table S3). Together, these data suggest that IDH1 R132Q-expressing chondrosarcoma models relied on upregulation of intra- and extra-cellular communication perhaps driven in part *via* direct CpG hypomethylation, as well as increased expression of oncogenes such as *MYC* and *EGFR* in mechanisms likely not directly dependent on altered DNA methylation.

### Epigenomic and transcriptomic features of mutant IDH1 glioma models

**Figure 4:**
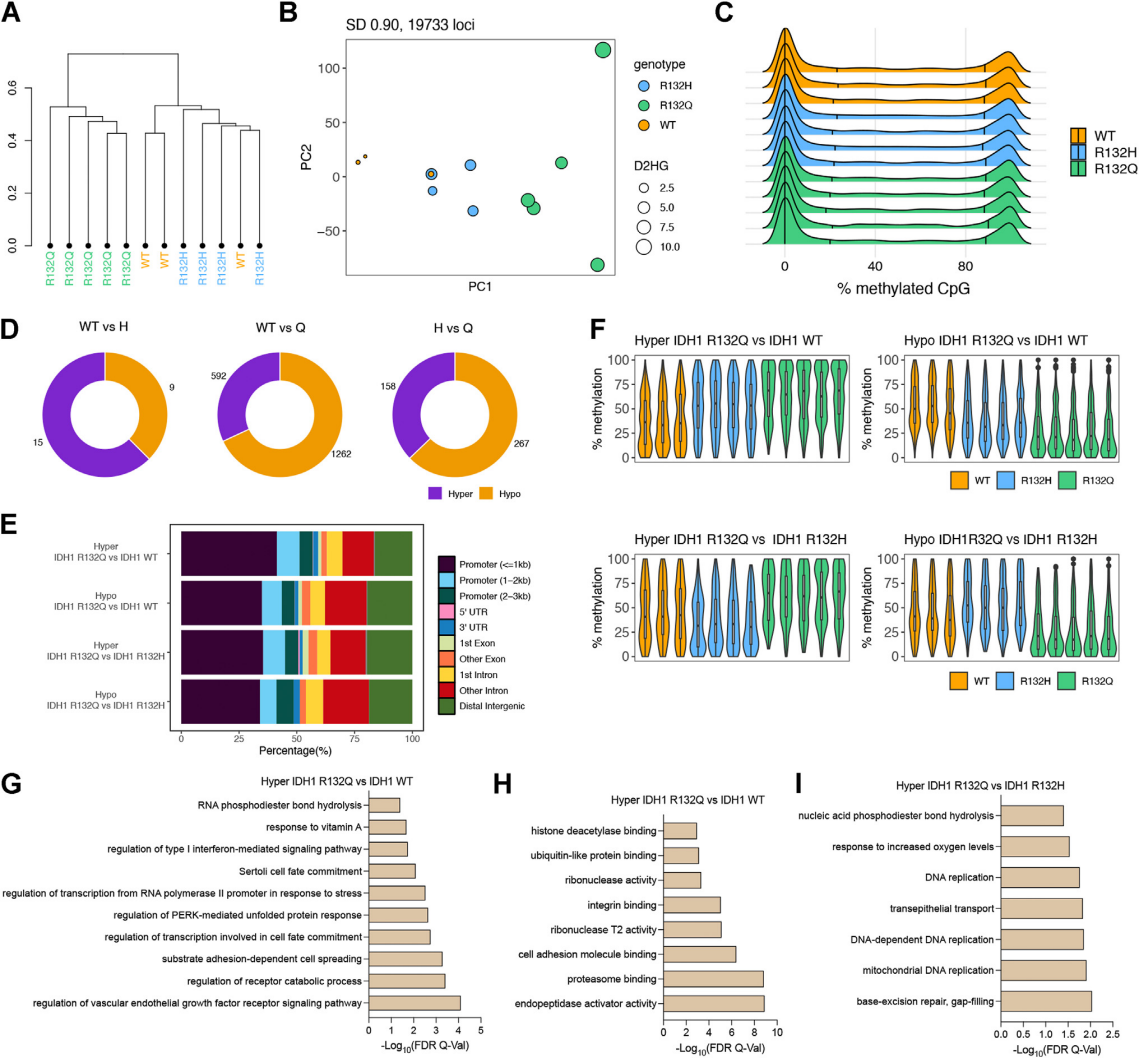
**IDH1 mutants exhibit a subtler influence on the methylomes of U87MG tumors.** Three of nine, four of nine, and five of nine (randomized selection for each) of the xenograft tumors that formed U87MG xenograft tumors upon IDH1 WT, R132H, or R132Q expression, respectively, were evaluated as biological replicates by RRBS. *A*, hierarchical clustering of the most variable CpG sites (S. D. > 0.90, n = 19,733) within the U87MG RRBS cohort. *B*, PCA plot of the most variable CpG sites (S. D. > 0.90) within the U87MG RRBS cohort. The size of each dot corresponds to the level of D2HG (nmol tumor) measured in each tumor sample. Colors indicate genotype. *C*, global methylation profiles of the U87MG RRBS tumor cohort showing percent methylation values of CpG sites for each sample. *Black vertical* lines indicate quantiles; colors indicate genotype. *D*, number of differentially methylated CpG sites (DMS, % methylation difference ≥ 10 and q-value < 0.05) are shown. Of note, RRBS data is filtered to include variable loci (S. D. > 10, n = 42,310) prior to differential methylation analysis. *E*, distribution of the genomic features for the hyper- and hypo-methylated loci for all comparisons. *F*, violin plots showing the distribution of percent (%) methylation of hypermethylated loci in IDH1 R132Q *versus* IDH1 WT (*top left*), hypomethylated loci in IDH1 R132Q *versus* IDH1 WT (*top right*), hypermethylated loci in IDH1 R132Q *versus* IDH1 R132Q (*bottom left*), and hypomethylated loci in IDH1 R132Q *versus* IDH1 R132H (*bottom right*). For clarity, samples not included in the pairwise statistical comparisons are also plotted. *G*–*I*, gene ontology enrichment (using GREAT toolbox) showing (*G*) enriched biological processes in hypermethylated (hyper) loci in IDH1 R132Q compared to IDH1 WT, (*H*) molecular function (MF) in IDH1 R132Q *versus* IDH1 WT, and (*I*) biological processes (BP) in hypermethylated loci in IDH1 R132Q compared to IDH1 R132H.

U87MG xenografts, which had more power than HT1080∗ xenografts due to higher tumor incidence, showed distinct clustering of IDH1 R132Q xenografts, with some clustering seen for WT and R132H tumors (Fig. 4*A*). PCA with overlaid D2HG levels showed better separation for the U87MG xenografts than HT1080∗ (Fig. 4*B*). Surprisingly, there was no observed increase in overall methylation among all CpG sites in IDH1 mutants compared to WT (Fig. 4*C*). Relative to the HT1080∗ xenografts, these mutant IDH1 U87MG xenografts had fewer differentially methylated CpG sites compared to WT (Fig. 4*D*). Therefore, we filtered the RRBS data and restricted our differential methylation analyses to variable CpG sites. After this, we found that a large percentage of the differentially methylated sites were hypomethylated (n = 1262) in IDH1 R132Q-expressing U87MG tumor xenografts compared to both WT and R132H (Fig. 4*F*). Trends in location of hypermethylation and hypomethylation in the glioma models were similar to chondrosarcoma models, with most alterations occurring near promoter regions followed by distal intergenic and other intron regions (Fig. 4*E*). Compared to HT1080∗ xenografts, U87MG xenografts had more hypomethylation and hypermethylation in regions outside the CpG islands and shores in addition to the CpG islands (Fig. S8).

### Pathways hypermethylated and downregulated in IDH1 R132Q glioma models

**Figure 5:**
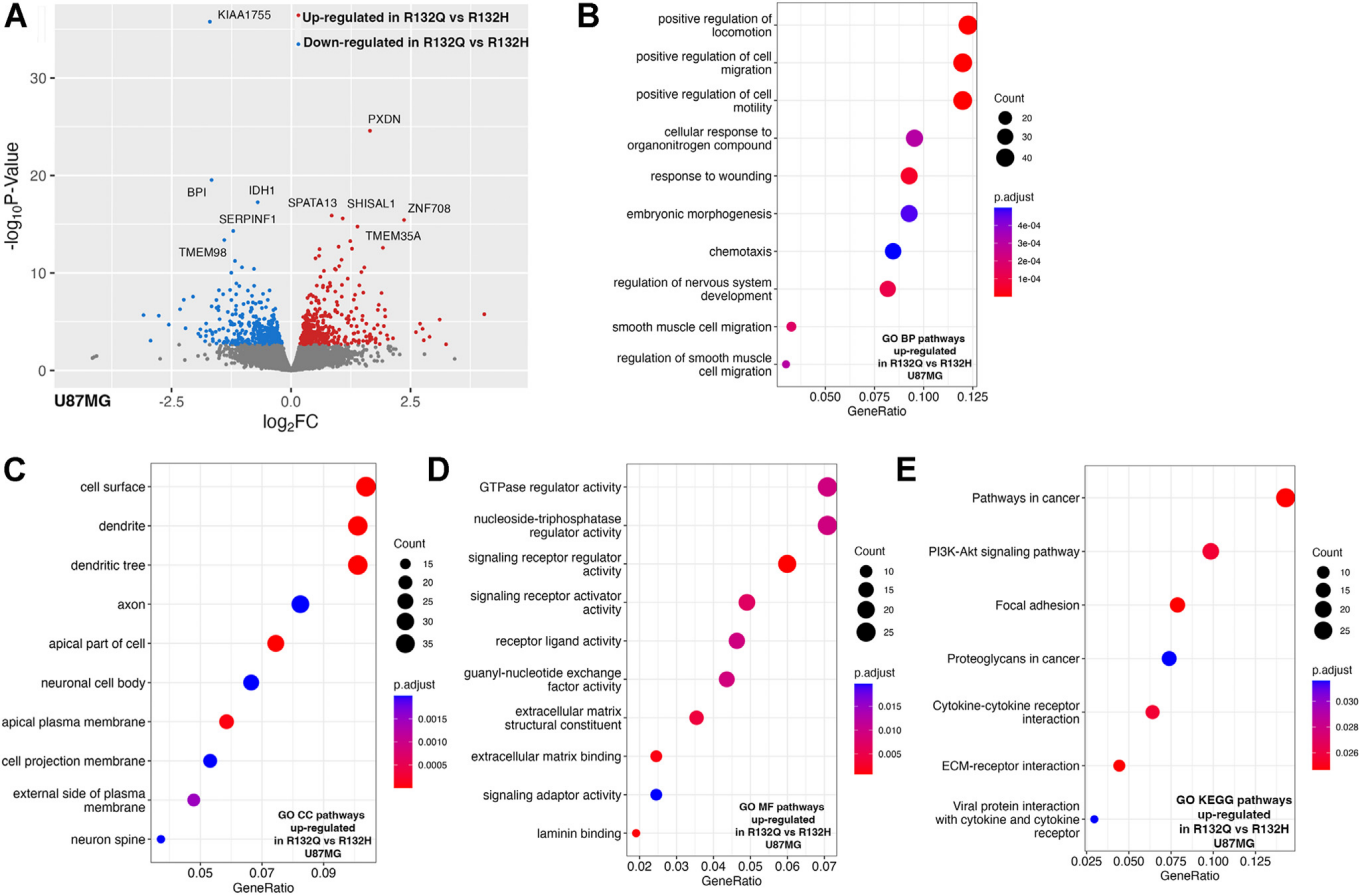
**Transcriptome analysis using RNAseq of U87MG xenograft tumors comparing IDH1 R132Q and IDH1 R132H.** Three of nine, four of nine, and five of nine (randomized selection for each) of the xenograft tumors that formed U87MG xenograft tumors upon IDH1 WT, R132H, or R132Q expression, respectively, were evaluated as biological replicates by RNAseq. Downregulated pathways refer to categories where most genes show decreased expression levels in R132Q *versus* R132H IDH1. Upregulated pathways refer to categories where most genes show increased expression levels in R132Q *versus* R132H IDH1. Number of genes is indicated in the count, with the *p*_adjusted_ value indicated by color. *A*, volcano plot of differentially expressed transcripts comparing expression of IDH1 R132Q *versus* IDH1 R132H. *B*, biological pathways (BP) upregulated in IDH1 R132Q *versus* IDH1 R132H. *C*, cellular component (CC) pathways upregulated in IDH1 R132Q *versus* IDH1 R132H. *D*, molecular function (MF) pathways upregulated in IDH1 R132Q *versus* IDH1 R132H. *E*, KEGG pathways upregulated in IDH1 R132Q *versus* IDH1 R132H.

RRBS analysis highlighted that regions associated with nucleic acid phosphodiester bond hydrolysis and DNA replication and repair were hypermethylated in IDH1 R132Q-expressing U87MG tumor xenografts *versus* R132H (Fig. 4*I*), reminiscent of DNA damage pathways hypermethylated in R132Q HT1080∗ tumors (Fig. 2*J*). This suggests that silencing DNA damage repair is likely an important strategy in both IDH1 R132Q-expressing tumor models. When comparing IDH1 R132Q glioma xenografts with WT, pathways associated with cell adhesion, integrin binding, and stress response were highlighted as hypermethylated (Fig. 4, *G* and *H*). Integrins, whose hypermethylation has been implicated in tumors (54), mediate cell adhesion to ECM and their activation can induce intracellular signaling cascades to affect cell growth, migration, differentiation, adhesion, and apoptosis. RNAseq analysis showed no pathways being significantly downregulated upon IDH1 R132Q expression *versus* R132H, though many similarities in downregulated pathways emerged for both mutants compared to WT, including pathways associated with chemotaxis, ECM organization, angiogenesis, and negative regulation of cell migration (Figs. 5, S12, and S13). Regions associated with the response to increased oxygen levels were also hypermethylated in R132Q U87MG tumors compared to R132H (Fig. 4*I*). Together, these data suggest that expression of both IDH1 mutants in glioma xenograft models share many transcriptionally down-regulated pathways, though IDH1 R132Q uniquely shows hypermethylation of DNA damage pathways.

### Pathways hypomethylated and upregulated in IDH1 R132Q glioma models

RNAseq analysis showed that there were many upregulated pathways that may have helped drive the larger, earlier-forming tumors observed in IDH1 R132Q U87MG xenografts compared to R132H, including cell migration, motility, cell signaling pathways. A comparison of upregulated pathways in R132Q *versus* WT xenografts recapitulated reliance on these pathways, with transcripts of genes related to cell migration, motility, and negative regulation of differentiation upregulated (Fig. S12). Compared to IDH1 R132H, expression of R132Q in U87MG xenografts was associated with an upregulation of motility and migration, and, perhaps surprisingly, pathways associated with neurons and neural development (Fig. 5, *B* and *C*). These pathways included genes representing the bone morphogenetic proteins (*BMP5, BMP7*), ephrin receptors (*EPHB2*), nephrin-like proteins (*KIRREL3*), semaphorins (*SEMA3A*), and the growth factor brain-derived neurotrophic factor *BDNF* (Table S4). Recent work has shown that neuronal migration and development mechanisms help drive the migration of invasive glioblastoma cells (55), with structural features of glioma migration including the formation of filopodia and the transport of integrins to the tips of these protrusions (56). Using scanning electron microscopy (SEM), we noticed evidence of migration in U87MG cells expressing IDH1 WT, R132H, and R132Q. Upon expression of WT IDH1, filopodia appeared to be the dominant feature of migration, while expression of IDH1 R132H and R132Q showed both filopodia and lamellipodia (Fig. S14, *A*–*C*). In particular, U87MG cells expressing IDH1 R132H appeared to have both filopodia and lamellipodia features, while cells expressing R132Q appeared to have a preference for lamellipodia formation (Fig. S14, *B* and *C*). Both filopodia and lamellipodia are critical for motility in cancer cells, with the former having a more exploratory role and the latter serving as a motor for movement (57). Though the lamellipodium organization pathway was not highlighted as significant in our GO analysis, all lamellipodium-related transcripts (*ABI1, ATP7A, ITGB1, S1PR1, SPATA13,* and *VAV3*) that were shown to be differentially expressed when comparing the mutants all had the same trend: they were significantly higher upon expression of IDH1 R132Q *versus* R132H in U87MG cells (Table S4). RNAseq analysis also showed significant upregulation of many integrins (*ITGA2, ITVAV, ITGB1, ITGA8*) upon expression of IDH1 R132Q *versus* R132H (Table S4). However, neither cell proliferation nor cell migration were significantly different upon expression of IDH1 WT, R132H, or R132Q, with a non-significant trend of increased migration in IDH1 WT-expressing cells *versus* mutant (Fig. S14, *D* and *E*). This suggests that these pathways may be more important in the tumor environment and that distinguishing filopodia *versus* lamellipodia features may not be suitably parsed in wound healing (scratch) assays.

Upregulation of the PI3K/AKT/mTOR pathway was apparent in IDH1 R132Q-expressing U87MG xenografts compared to R132H; PI3K/AKT/mTOR signaling *via* upregulation of *EGFR*, interleukin 6 (*IL6*), and janus kinase 1 (*JAK1*) were all significantly upregulated in R132Q U87MG xenografts (Table S4). Previously, the PI3K/AKT/mTOR pathway has been shown to be downregulated in R132H-driven tumors and tumor models. It was suggested that D2HG production inhibited AKT phosphorylation (58, 59), though upregulation in our R132Q xenografts suggests a different mechanism may be at play. *EGFR*, *IL6*, and *JAK1* upregulation also suggests that R132Q expression correlates with JAK-STAT pathway activation, and we observed a (nonsignificant) trend of increased *STAT3* expression (Table S4). We used Western immunoblotting of our U87MG cell lines to assess relative protein expression and phosphorylation of several members of these pathways. We observed evidence of a trend of increased phosphorylated mTOR (p-MTOR) in U87MG cells expressing R132Q *versus* R132H, as well as in phosphorylated EGFR (p-EGFR) to a much smaller degree (Fig. S15). However, phosphorylated PI3K was not detected, and phosphorylated AKT (p-AKT) was slightly decreased upon expression of IDH1 R132Q, while phosphorylated STAT3 (p-STAT3) showed essentially no change (Fig. S15). The PI3K/AKT/mTOR pathway, through activation of growth factor receptors like EGFR and integrins, can also activate MDM2, which in turn can degrade p53 (60). Notably, IDH1 R132Q-expressing U87MG tumor xenografts had significantly increased transcripts of many integrins as described, as well as *EGFR* and *MDM2* (Table S4) compared to R132H. When comparing IDH1 R132Q to WT, all transcripts except *MDM2* were also significantly increased (and *MDM2* transcripts trended upwards), and *TP53* and *TP53I3* were significantly downregulated (Table S4).

Notably, n-Ras (*NRAS*), as well as transforming growth factor β-1 (*TGFB1*) and transforming growth factor α (*TGFA*), which are downstream targets of n-Ras, were significantly upregulated in IDH1 R132Q-expressing U87MG xenografts compared to WT (Table S4 and Fig. S12), supportive of activation of cancer growth and metastasis pathways (61). Many of these oncogenes are also implicated in focal adhesion, which helps regulate cell growth, migration, and ECM remodeling, and evidence of the importance of these pathways were supported by our observed upregulation of c-Jun (*JUN*), Rho associated coiled-coil containing protein kinase 2 (*ROCK2*), and several collagen genes upon R132Q expression compared to WT (Table S4). Importantly, no significant upregulation in these oncogenes that IDH1 R132Q appeared reliant upon was seen when comparing R132H *versus* WT-expressing U87MG xenografts (Fig. S13 and Table S4). Together, these data highlight likely a non-methylation-based reliance of IDH1 R132Q-expressing U87MG xenografts on the activation of PI3K/AKT/mTOR, EGFR, and Ras pathways and deactivation of p53 to lead to increased cell growth and migration.

## Discussion

We expected that higher D2HG levels resulting from more efficient IDH1 R132Q catalysis would result in increased DNA methylation, but instead we observed primarily genome hypomethylation upon expression of IDH1 R132Q *versus* R132H in our xenograft models. Increased hypermethylation and hypomethylation are well-documented in R132H IDH1-mutated tumor models and tumor tissue (3, 9, 28, 62, 63, 64, 65, 66, 67, 68), though the mechanisms and consequences of hypomethylation are not understood (62, 63, 64, 65). Interestingly, malignant progression of mutant IDH1-driven lower grade gliomas is associated with partial DNA demethylation *via* an inability of rapidly proliferating cells to maintain methylation (69). Upon this passive demethylation, tumors had activated PI3K/AKT/mTOR and cell cycle pathways *via* mutation and altered transcript levels (69), which is in contrast to the downregulation of MAPK, PI3K/AKT/mTOR, and EGFR pathways normally associated with IDH1 R132H-driven tumors (4). In fact, *EGFR* amplification and R132H IDH1 mutations are uncommon or even mutually exclusive (70, 71, 72). Others have reported that faster progressing mutant IDH1-driven astrocytomas had increased copy numbers in *EGFR*, *MYC*, *MET*, and *CDK6* compared to slower-progressing patients (73). Interestingly, our own assessment of the Brain Lower Grade Glioma TCGA PanCancer Atlas dataset (13) highlighted that low expression of TP53I3 and high expression of IL6 and MYC was associated with worse survival in mutant IDH1 tumors but not WT IDH1 (Fig. S16). DNA hypomethylation can also result from accumulation of 8-oxo-2′-deoxyguanosine, the product of the interaction between reactive oxygen species (ROS) and guanosine. This type of DNA damage can lead to DNA demethylation through pathways including inability of cytosines adjacent to 8-oxo-2′-deoxyguanosine to be methylated, or from ROS inducing TET activity (74, 75, 76, 77). Interestingly, we found that cellular ROS levels were higher upon expression of IDH1 R132Q in U87MG cells compared to R132H (Fig. S17). As genes associated with DNA damage repair and regions associated with the response to increased oxygen levels were hypomethylated (Fig. 4*I*), this may be an important mechanism to explore in the future. Overall, our observations of increased hypomethylation, larger tumor size, activation of PI3K/AKT/mTOR, EGFR, Wnt, and Ras pathways, and downregulation of p53 pathway upon IDH1 R132Q expression supports these findings, suggesting that the R132Q mutation somehow confers features reminiscent of more aggressive, typically non-IDH1-driven tumors through mechanisms not likely directly involving CpG hypermethylation.

We found that correlation between DNA methylation and gene expression was relatively poor, and others have reported the same finding, citing that locations that acquire hypermethylation may often affect regions that already are transcriptionally less active (64). Of course, changes in D2HG-driven histone methylation as well as indirect changes in protein activation *via* phosphorylation will also affect the transcriptome (4). Our limited power in the WT IDH1 HT1080∗ tumors due to lack of tumor formation in 7/9 mice likely complicated analysis, as one of the two WT tumors was unusually large (Table S1).

The decreased methylation in mutant IDH1 U87MG xenografts compared to WT in our initial data processing was surprising. A possible reason for this is due to the low passage number in the U87MG cells; after successfully introducing the mutations, the U87MG cells were injected into mice at a passage number range of 6 to 12, while HT1080∗ lines were used at passage 21. We (9) and others (74) have shown that the degree of methylation changes over time, with high passage number associated with increased hypermethylation. However, deletion of IDH1 mutations have been reported both *in vitro* and *in vivo* (67), and indeed, further expansion of our U87MG line, but not our HT1080∗, led to the loss of expression of the R132Q mutation. To ensure that IDH1 R132Q was still expressed and to allow head-to-head comparisons of U87MG cells at similar passage number, xenograft injections occurred at this lower passage despite suboptimal conditions for RRBS analysis. Despite this challenge, a trend unique to IDH1 R132Q emerged in both chondrosarcoma and glioma xenografts – hypermethylation of pathways associated with DNA damage. There is support that hypermethylation of DNA damage pathways is unique to the IDH1 R132Q mutant; H3 histone hypermethylation studies with *in vivo* IDH1 R132H-expressing tumor models have suggested up-regulation of DNA damage response and cell cycle control (75).

Altered Wnt signaling can ultimately result in dysregulation of differentiation and morphogenesis to help drive tumor progression (48, 76, 77, 78, 79), with this pathway emerging as a possible therapeutic target due to its important role in glioma invasiveness and stemness (80). Hypermethylation and/or down-regulation of Wnt signaling modulators and pathway members to result in aberrant Wnt signaling has been reported as a consequence of IDH1 R132H expression by us (9) and others (64, 81, 82, 83). Hypermethylation of Wnt signaling in general has been implicated in a variety of cancers (77, 84), including hypermethylation of *WNT9A* and *FZD9* genes (76, 85). WNT9A has tumor suppressor features in that it drives the differentiation of chondrocytes (78) and can inhibit cell proliferation (79). We found here that alterations in the Wnt pathway represented another persistent phenotype in our xenograft models, with primarily hypermethylation (and some hypomethylation) of gene pathway members upon expression of IDH1 R132Q in chondrosarcoma models. Notably, transcripts of many Wnt pathway members were significantly both up- and down-regulated upon IDH1 R132Q expression in both xenograft models (Tables S3 and S4), suggesting that non-epigenetic changes may also alter the regulation of this pathway.

Finding distinct methylation and transcription patterns when comparing HT1080∗ and U87MG xenografts was unsurprising, as this has been reported in tumors and tumor models of mutant IDH1-driven cancers. HT1080 and U87MG lines represent different cells of origin, and the HT1080∗ cells used here originated from an IDH1^R132C/+^ background that likely had epigenetic changes prior to deletion of the R132C allele. HT1080∗ cells also have only half the endogenous WT allele amount compared to U87MG cells, and there have been differences reported between hemizygous and heterozygous tumor models (86). Interestingly, there is a clear mutation selection bias in gliomas (IDH1 R132H) *versus* chondrosarcomas (IDH1 R132C) (13), and to date, IDH1 R132Q has only been reported in chondrosarcomas (11). Though differences in catalytic profiles between IDH1 R132H and R132C were not nearly as profound as that seen in R132Q, we showed previously that R132C has a ∼3-fold increase in *k*_cat_ compared to R132H (21). Thus, this preference for the R132C mutation and allowance of R132Q in chondrosarcomas *versus* R132H in gliomas raises the possibility that glioma cells could be more susceptible to D2HG toxicity and thus prefer the lower D2HG levels associated with R132H. There is also debate about which mutant IDH-driven tumor type is associated with more hypermethylation. Some have shown that IDH-driven gliomas are more hypermethylated than AML, melanomas, and cholangiocarcinomas (65), though others report AML as having the highest methylation (62). Understanding the unique DNA and histone methylation features among different tumor tissue and among different IDH1 and IDH2 mutants remain important areas of research. An important long-term goal would be a comparison of the methylation and transcription profiles of tumor models of less common but physiologically relevant IDH1 mutants, especially those we and others have shown to have distinct D2HG levels and kinetic profiles (20, 21, 22).

We also have previously reported that IDH1 R132Q uniquely maintains some conventional reaction activity (21, 23). It has been shown that overexpression of WT IDH1 has been implicated in glioblastomas, leading to increased tumor cell growth, metabolic reprogramming including lipid biosynthesis, and decreased survival in *in vivo* models (87). It is possible that this retention of conventional activity of IDH1 R132Q may also help drive some of the observed phenotypes. However, we note that any changes in ICT and αKG concentrations in the xenografts are minimal when comparing R132H and R132Q (Table S5). The conventional reaction catalyzed by IDH1 R132Q may have only a minor role physiologically; the high *K*_m, ICT_ (low mM range) means that under most physiological conditions the conventional reaction is likely inefficient or essentially negligible, though there may be important consequences upon inhibitor binding (22, 23).

In summary, we leveraged steady-state kinetics, metabolic profiling, epigenomics, and transcriptomics to connect the biophysical features of tumor-driving IDH1 mutations to *in vivo* phenotypes in tumor models. Identification of an IDH1 mutation with distinctly robust neomorphic activity provided an ideal opportunity to establish the consequences of IDH1 mutation with low *versus* high catalytic efficiency for D2HG production. Our findings showed that higher catalytic activity afforded by IDH1 R132Q *versus* R132H led to increased D2HG levels in cells, tumor tissue, and sera, and larger tumors upon expression of this highly kinetically active mutant, despite its lower expression. Further, IDH1 R132Q expression led to activation of several pro-tumor pathways, including those associated with EGFR and PI3K signaling reminiscent of lower grade mutant IDH1 tumors that progress to higher grade. Our work highlights a potential ability of D2HG levels to tune phenotype severity through mechanisms beyond increased DNA hypermethylation.

## Experimental procedures

### Chemicals and reagents

β-Nicotinamide adenine dinucleotide phosphate reduced trisodium salt (NADPH), β-nicotinamide adenine dinucleotide phosphate disodium salt (NADP^+^) tris(2-carboxyethyl)phosphine) (TCEP), agarose, *L*-norvaline, methoxyamine hydrochloride, *N*-tert-butyldimethylsilyl-*N-*methyltrifluoroacetamide, and 100 kD Amicon centrifugal filters were purchased from Millipore Sigma (Burlington, MA). Triton X-100, magnesium chloride (MgCl_2_), α-ketoglutaric acid sodium salt (αKG), DL-isocitric acid trisodium salt hydrate, dithiothreitol (DTT), isopropyl 1-thio-β-D-galactopyranoside (IPTG), Pierce protease inhibitor tablets, chloroform, isopropanol, PEI STAR transfection reagent, chloroquine diphosphate, T-PER Tissue Protein Extraction Reagent, Pierce ECL Western blotting substrate, *N*-acetyl-cysteine, *N*-acetyl-cysteine, cumene hydroperoxide hexamethyldisilazane (HMDS), 16% paraformaldehyde, DH5α *E. coli* strain, PCR *mycoplasma* detection kits, and Pierce RIPA buffer (catalog # 89900, lot ZD388406) were purchased from Thermo Fisher Scientific (Waltham, MA). *B*-mercaptoethanol (BME) was bought from MP Biomedicals (Santa Ana, CA). Nickel-nitrilotriacetic acid (Ni-NTA) resin, QIAzol Lysis Reagent, and EpiTect Fast DNA Bisulfite Kit (# 59824) were obtained from Qiagen (Valencia, CA). Pre-cast 4 to 20% and 4 to 12% stain-free gels, polyvinylidene fluoride (PVDF) membranes, Precision Plus protein dual color ladder, and 4X Laemmli sample buffer were purchased from Bio-Rad Laboratories (Hercules, CA). High glucose Dulbecco’s Modified Eagle's Medium (DMEM), phosphate-buffered saline (PBS), penicillin-streptomycin-glutamine (PSG) 100X, *L*-glutamine, and TrypLE express enzyme were purchased from Gibco (Grand Island, NY). Fetal bovine serum (FBS) was obtained from Cytiva (Marlborough, MA). Matrigel (lot 354263) and 45 μM vacuum filters were purchased from Corning (Corning, NY). MEGAPREP 3 kit was purchased from Bioland Scientific (Paramount, CA). GentleMACS M Tubes were purchased from Miltenyi Biotec (San Diego, CA). Aqueous 10% glutaraldehyde and 4% osmium tetroxide (OsO_4_) aqueous solution was purchased from Electron Microscopy Sciences (Hatfield, PA). A KAPA HiFi Hot Start Ready Mix PCR kit and KAPA Library Quant Kit ABI Prism qPCR Mix was purchased from Roche Sequencing Store (Boston, MA). HEK293T Lenti-X cells were purchased from Clontech (Mountain View, CA). Plasmids pVSVG, pRev, and pGal/Pol were purchased from Addgene (Watertown, MA). U87MG cells were purchased from ATCC (Manassas, VA). BD microtainer SST tubes were purchased from BD (Franklin Lakes, NJ). TaqaI and MspI were purchased from NEB (Ipswich, MA). A ROS-Glo H_2_O_2_ ROS and CellTiter-Glo Luminscent Cell Viability assay kits was purchased from Promega (Madison, WI).

Antibodies were purchased as follows: HA-tag (catalog #PA1-985 lots XG358301 and YL378360) was purchased from ThermoFisher (Waltham, MA); HRP-rabbit (catalog # PI32460 lot XG356768), and HRP-mouse (catalog # NC0338742 lot XD347166) were purchased from Invitrogen (Waltham, MA). GAPDH antibody (catalog # MAB374 lot 3988901, catalog # A21994 lot 3017349, and catalog # sc-47724 lot C1023)) were purchased from Sigma-Aldrich, Invitrogen (Waltham, MA), and Santa Cruz Biotechnology (Dallas, TX), respectively. Akt (catalog # 9272S lot 30), p-Akt (catalog # 9271S lot 15), EGFR (catalog # 4267S lot 24), p-EGFR (catalog # 3777T lot 17), mTOR (catalog # 2983S lot 21), p-mTOR (catalog # 2971S lot 28), STAT3 (catalog # 12640T lot 7), p-PI3K (catalog # 4228T lot 7) and p-STAT3 (catalog # 9145T lot 43) were purchased from Cell Signaling Technology (Danvers, MA). Lambda protein phosphatase was purchased from NEB (catalog #M0303S, lot 10258557, Ipswich, MA).

Antibody specificity was indicated by the manufacturer as follows: anti-HA: https://www.thermofisher.com/antibody/product/HA-Tag-Antibody-Polyclonal/PA1-985; anti-GAPDH: https://www.sigmaaldrich.com/US/en/product/sigma/zrb374?utm_source=google&utm_medium=cpc&utm_campaign=21466469979&utm_content=165394553472&gbraid=0AAAAAD8kLQTZwO5PpL17tUVnwYCgCcXeU&gclid=CjwKCAjwuMC2BhA7EiwAmJKRrErbBim_mGameNXWwQD9wslg_mtgFZFLq_QqOH4yaQb-UVjrrc8w_RoC0jUQAvD_BwE; anti-Akt: https://www.cellsignal.com/products/primary-antibodies/akt-antibody/9272?qs=keyword_redirect&qt=9272S; anti-p-Akt: https://www.cellsignal.com/products/primary-antibodies/phospho-akt-ser473-antibody/9271; anti-EGFR: https://www.cellsignal.com/products/primary-antibodies/egf-receptor-d38b1-xp-rabbit-mab/4267; anti-p-EGFR: https://www.cellsignal.com/products/primary-antibodies/phospho-egf-receptor-tyr1068-d7a5-xp-rabbit-mab/3777; anti-mTOR: https://www.cellsignal.com/products/primary-antibodies/mtor-7c10-rabbit-mab/2983; anti-p-mTOR:https://www.cellsignal.com/products/primary-antibodies/phospho-mtor-ser2448-antibody/2971; anti-p-PI3K: https://www.cellsignal.com/products/primary-antibodies/phospho-pi3-kinase-p85-tyr458-p55-tyr199-antibody/4228; anti-STAT3: https://www.cellsignal.com/products/primary-antibodies/stat3-d3z2g-rabbit-mab/12640; anti-STAT3: https://www.cellsignal.com/products/primary-antibodies/stat3-d3z2g-rabbit-mab/12640; anti-p-STAT3: https://www.cellsignal.com/products/primary-antibodies/phospho-stat3-tyr705-d3a7-xp-rabbit-mab/9145; anti-rabbit IgG: https://www.cellsignal.com/products/secondary-antibodies/anti-rabbit-igg-hrp-linked-antibody/7074; and anti-mouse IgG: https://www.cellsignal.com/products/secondary-antibodies/anti-mouse-igg-hrp-linked-antibody/7076.

### Protein expression, and purification

Human IDH1 WT, R132H, and R132Q cDNA constructs in a pET-28b(+) plasmid were transformed in *E. coli* BL21 Gold DE3 cells. After incubation in 0.5 to 1 L of terrific broth containing 30 μg/ml of kanamycin (37 °C, 200 rpm), induction began with 1 mM IPTG (final concentration) after briefly cooling cultures to 25 °C after reaching an A_600_ of 0.9 to 1.2. After an 18 h incubation (19 °C, 130 rpm), cell pellets were harvested and resuspended in lysis buffer (20 mM Tris pH 7.5 at 4 °C, 500 mM NaCl, 0.1% NaCl, 0.1% Triton X-100, and one protease inhibitor tablet) for cell lysis *via* sonication. Crude lysates were clarified *via* centrifugation at 12,000 rpm for 1 h. The resulting lysate was loaded on to a pre-equilibrated Ni-NTA column, followed by 150 ml of wash buffer (20 mM Tris pH 7.5 at 4 °C, 500 mM NaCl, 15 mM imidazole, 5 mM BME). Protein was then eluted with elution buffer (50 mM Tris pH 7.5 at 4 °C, 500 mM NaCl, 500 mM imidazole, 5% glycerol, 10 mM BME). Protein was concentrated if needed and dialyzed overnight in 50 mM Tris pH 7.5 at 4 °C, 100 mM NaCl, 20% glycerol, and 1 mM DTT. Greater than 95% purity was ensured *via* SDS-PAGE analysis, and IDH1 was flash frozen using liquid nitrogen, and stored at −80 °C. All kinetic analysis was performed < 1 month from cell pelleting.

### Enzyme kinetic analysis

An Agilent Cary UV/Vis 3500 spectrophotometer (Santa Clara, CA) was used to perform steady-state kinetic assays at 37 °C. To allow heterodimers to form, a 1:1 mixture (200 nM final concentration of both WT and mutant IDH1) was incubated on ice for 1 h after gentle mixing. However, we note that a mixture of homodimers and heterodimers, depending on their binding equilibria, may exist. To measure the conventional reaction (conversion of ICT to αKG), a cuvette containing IDH1 assay buffer (50 mM Tris, pH 7.5 at 37 °C, 150 mM NaCl, 10 mM MgCl_2_, 1 mM DTT), and the mixture of WT and mutant (R132H or R132Q) IDH1 (200 nM) were preincubated for 3 min at 37 °C. NADP^+^ (200 μM) and varying concentrations of ICT were added to initiate the reactions and the change in absorbance due to NADPH formation was monitored at 340 nm. The same conditions were used for the neomorphic reaction (conversion of αKG to D2HG) except NADPH and αKG were used to initiate the reaction and the consumption of NADPH was monitored. The pH of αKG was adjusted to 7.0 prior to use. The slope of the linear range of the change in absorbance over time was calculated and converted to nanomolar NADPH using the molar extinction coefficient for NADPH of 6.22 cm^−1^ mM^−1^ to determine *k*_obs_ (*i.e.* nM NADPH/nM enzyme s^−1^) at each substrate concentration. Each *k*_obs_ was fit to the Michaelis-Menten equation in GraphPad Prism (GraphPad Software) to calculate *k*_cat_ and *K*_m_. In all cases, two biological replicates (two protein preparations) were used to measure *k*_obs_ over multiple days, ensuring both preparations were tested at the full range of concentrations of substrate to ensure batch-to-batch reproducibility. Kinetic parameters are reported as standard error (S.E.) resulting from deviation from the mathematical fit of the equation.

### Cell line generation and culturing

LEIH-IDH1-R132H, LEIH-IDH1-WT, and LEIH-IDH1-EV plasmids conferring hygromycin resistance and containing a *C*-terminal HA-tag were obtained from William Kaelin Jr (Dana Farber Cancer Institute). The LEIH vector has an EF1a promoter for high expression. We used site-directed mutagenesis to introduce the R132Q mutation by removing the IDH1 WT insert from the LEIH vector, inserting this into a pET17b vector and performing mutagenesis using a KAPA HiFi Hot Start Ready Mix PCR kit using the following mutagenesis primers: forward -- 5′-CTGTATTGATCCCCATAAGCATGCTGACCTATGATGATAGGTTTTACC; reverse – 5′-GGTAAAACCTATCATCATAGGTCAGCATGCTTATGGGGATCAATACAG. The IDH1 insert was then returned to the LEIH vector. All LEIH plasmids confer kanamycin resistance in bacteria and were amplified in the DH5α *E. coli* strain. Plasmids were purified using MEGAPREP 3. Plasmid whole genome sequencing was performed by Retrogen Inc. (San Diego, CA). The hygromycin-resistant genes linked to IDH1 *via* the internal ribosome entry site (IRES) allow its continuous growth in a low concentration of hygromycin, which was supplemented to the media at the selection dose of 100 μg/ml (final concentration). Only cells that survived were selected, and fresh media was added to flasks with a concentration of 60 μg/ml hygromycin to prevent silencing of the mutation over time. Lentiviral particles were produced in HEK293T Lenti-X cells. Cells (5 × 10^6^) were seeded in a 15 cm culture flask and allowed to reach ∼70% confluency. LEIH vectors of interest were co-transfected with the lentiviral packaging, envelope, and infection system plasmids pVSVG, pRev, and pGal/Pol. The transfected cells were incubated for 16 h, and the media was collected and passed through a 45 μM vacuum filter. To concentrate the viral particles, centrifugation of the sample in a 100kD Amicon centrifugal filter was used, and the viral particles were stored at −80 °C.

U87MG cells were purchased from ATCC, which performs authentication and quality control tests. HT1080 cells, which contain an endogenous R132C mutation (IDH1^R132C/+^) were modified by Kun-Liang Guan (University of California, San Diego) to knock-out the mutant allele to generate (IDH1^−/+^) (88), here notated as HT1080∗, which were then provided to us. Sequencing was used to confirm both IDH1 alleles, and wells were assessed by phenotypic examination. Normal human astrocytes (NHA) were obtained from Russ Pieper (University of California, San Francisco), and C28 cells were obtained from Johnathan Trent (University of Miami). Cells were assessed by phenotypic examination. Routine *mycoplasma* testing was performed as infrequently as every 6 months and as frequently as monthly, and always before experiments (*e.g.* xenograft generation, RRBS or RNAseq analysis) to ensure no contamination was present. Cells stably over-expressing IDH1 WT, R132H, or R132Q were prepared in each of these four cell lines by culturing cells in T75 flasks and allowing them to reach ∼60% confluency in DMEM supplemented with 10% FBS. Subsequently, the media was replaced with fresh DMEM, and 20 μl of a fresh aliquot of LEIH virus was added directly to the flasks. After 72 h past transfection, media was replaced with DMEM containing 10% FBS and 60 μg/ml hygromycin for cell selection. To assess the success of transfection, we extracted the total protein from the mutant cells and conducted western immunoblotting using a recombinant anti-HA tag antibody.

For routine culturing, the U87MG, HT1080∗, NHA, and C28 cells were cultured in DMEM containing 10% FBS and hygromycin at 60 μg/ml (final concentration). HT1080 and U87MG cells were seeded in the concentration of 2 × 10^5^ of U87MG in a T75 flask. Cells were allowed to reach ∼60% confluency before splitting, with fresh media was added every 48 h. All cell lines used were negative for *mycoplasma* contamination per our PCR-based testing both upon first use of the cells, as well as our routine testing that occurred no less frequently than once per 6-month interval or before introduction into animals.

### Ethics statement

All animal experiments, procedures, and maintenance were conducted in accordance with the San Diego State University (SDSU) Institutional Animal Care and Use Committee guidelines (protocol approval number 22-06-007s).

### Animals

Female athymic nude mice (6–8 weeks old) were purchased from Jackson Labs (Sacramento, CA). Female mice were selected based on previously reported protocols for mutant IDH1 xenograft generation (89). Mice underwent a mandatory period of 1-week acclimatization at the SDSU Animal Facility. The animals were housed in individually ventilated cages (ABSL2) with a maximum of four mice per cage. The mice housing environment was maintained throughout the study with a temperature range of 22 to 25 °C, humidity between 40 to 60%, and a light cycle of 12 h of light and 12 h of darkness. Cages, bedding, water, and food were individually irradiated for sterility. The selection of nine mice per group (27 mice total) was to achieve a conservative effect size of 0.4, as calculated by power analysis.

### Xenograft generation and analysis

Mice were randomized to receive xenograft injections. For xenografts, 2 × 10^6^ U87MG IDH1 WT, IDH1 R132H, or IDH1 R132Q cells suspended in Matrigel and PBS (1:1) or 2 × 10^6^ HT1080∗ IDH1 WT, IDH1 R132H, or IDH1 R132Q cells suspended in PBS were implanted subcutaneously into the left flanks of 6–8-week-old female athymic Nu/Nu mice. For each IDH1 expression condition (WT, R132H, or R132Q), nine mice were used (27 mice total) to achieve a conservative effect size of 0.4, as calculated by power analysis. Based on previous work on generating xenografts with HT1080 cells (90, 91), Matrigel was not used for the HT1080∗ cells. Vehicle controls (1:1 Matrigel/PBS or PBS alone) were injected subcutaneously into the corresponding right flanks. Twice weekly, mice were weighed, and tumor volume was measured blinded with a Vernier caliper and calculated using the following equation: Tumor Volume (mm^3^) = ½ length^2^ (long axis) × width (short axis). Mice were sacrificed once tumors reached 20 mm in length or 120 days of growth. Blood was collected in BD microtainer SST tubes and processed according to manufacturer's protocol. Tumors were weighed. Serum and tumor tissue were flash-frozen for downstream analysis. Tumor measurements were made fully anonymized.

### Western immunoblotting

Protein was extracted from tumor tissue by combining 100 to 200 mg of tumor tissue with 500 μl of T-PER in gentleMACS M tubes, and samples were dissociated mechanically using a gentleMACS Dissociator (Miltenyi Biotec). All tubes were incubated on ice for 15 min post-tissue homogenization. After incubation, samples were transferred to autoclaved 1.7 ml microtubes and clarified by centrifugation at 13,000 rpm for 20 min. The supernatant was then transferred to fresh 1.7 ml microtubes and stored at −20 °C. Protein was extracted from cell lines by culturing cells in T75 flasks and allowed them to reach ∼60% confluency in DMEM + 10% FBS. Cells were washed twice with PBS and detached from the flask using TrypLE Express enzyme. The enzyme activity was neutralized using complete media (1:1). Cells were transferred to a 15 ml conical tube and clarified using centrifugation at 1000 rpm for 10 min, after which the supernatant was discarded. Pierce RIPA buffer treated with 0.5 mM PMSF or homemade RIPA buffer (200 μl of 10 mM Tris pH 8.0, 140 nM NaCl, 1 mM EDTA, 0.5 mM EGTA, 1% Triton X-100, 0.1% sodium deoxycholate, and 0.1% SDS) treated with a Pierce Protease Inhibitor Mini Tablet was added to the cell pellet supplemented and incubated on ice for 15 to 30 min. In the case of phospho-antibody westerns, lambda protein phosphatase was added to serve as negative controls for phosphorylation assessment. The cell lysates were then transferred to an autoclaved 1.7 ml microtubes and clarified by centrifugation at 3000 rpm for 20 min. All cell line protein samples were stored at −20 °C. In all cases, protein samples were normalized to 20 μg/ml of protein using a BCA assay in 1X NuPAGE LDS sample buffer and Bond-Breaker TCEP solution, and incubated at 95 °C for 10 min. Protein samples were loaded to a NuPAGE 4 to 12% bis-tris precast gel for sample separation at 130 V for 90 min following a pre-electrophoresis at 20 V for 10 min, or to a Mini_protean TGX 4 to 20% precast gel at 120 V for 60 min following pre-electrophoresis. Protein samples were transferred to PVDF Immobilon-FL 0.45 μm membrane using an XCell SureLock Blot Module for 60 min at 1.0 A, 30 V, or using a Transblot Turbo for 7 min at 25 V, 2.5 A. Following blocking membranes in 5% skim milk in 0.1% TBST, or 5% BSA in 0.1% TBST the following antibody concentrations were used: anti-HA-tag (1:5000), γ-H2A.X (1:1000), GAPDH (1:6000), (Akt 1:1000), p-Akt (1:1000), EGFR (1:1000), p-EGFR (1:1000), mTOR (1:1000), p-mTOR (1:1000), p-PI3K (1:1000), STAT3 (1:1000), and p-STAT3 (1:1000). Primary antibodies were incubated overnight at 4 °C, and then the blots were washed and secondary antibodies were incubated for 1h at room temperature at the following concentrations: goat anti-rabbit IgG (H+L) HRP (1:2000) and anti-mouse IgG, HRP-linked (1:2000). Following washing in TBST, membranes were treated with SuperSignal West Pico PLUS Chemiluminescent Substrate, SuperSignal West Femto Maximum Sensitivity Substrate, or Clarity Western ECL Substrate for imaging on an iBright CL1000 Imaging Systems (ThermoFisher) or a ChemiDoc MP imaging system (BioRad). When necessary, the blots were stripped using the OneMinute Advance Western Blot Stripping Buffer per manufacturer's instructions (GM Biosciences). Protein expression levels were normalized to GAPDH.

### Metabolite analysis by mass spectrometry

Three biological replicates of U87MG, HT1080∗, C28, and NHA cells were allowed to reach ∼60% confluency in DMEM supplemented with 10% FBS. After washing the cells three times with cold PBS, 0.45 ml of 20 μM *L*-norvaline in 50% methanol (50% v/v in water) was added to serve as an internal standard. Flasks were incubated on dry ice for 30 min and then thawed on ice for 10 min. Cells were lifted from the flask using a cell scraper and transferred to an microtube tube. For mouse studies, for each biological replicate, ∼20 mg of each tumor xenograft was cut and weighed, and serum was collected. All samples were flash frozen in polypropylene tubes. All cell, tumor, and serum samples were shipped in dry ice to Sanford Burnham Prebys Medical Discovery Institute Protein Production and Analysis Facility to perform metabolite measurements. Dried methanol extracts were first derivatized by adding 50 μl of 20 mg/ml methoxyamine hydrochloride prepared in dry pyridine and incubated for 20 min at 80 °C. After cooling, 50 μl of *N*-tert-butyldimethylsilyl-*N-*methyltrifluoroacetamide was added, and samples were re-incubated for 60 min at 80 °C before centrifugation for 5 min at 14,000 rpm at 4 °C. The supernatant was transferred to an autosampler vial for gas chromatography-mass spectrometry (GC-MS) analysis. A TSQ 9610 GC-MS/MS (Thermo Scientific) with an injection temperature of 250 °C, injection split ratio 1/10, and injection volume 0.5 μl were used. GC oven temperature started at 130 °C for 4 min, rose to 243 °C at 6 °C/min and to 280 °C at 60 °C/min with a final hold at this temperature for 2 min. The GC flow rate with helium carrier gas was 50 cm/s, and a GC 15 m × 0.25 mm × 0.25 μm SHRXI-5 ms column was used (Shimadzu, Columbia, MA). GC-MS interface temperature was 300 °C, and the ion source temperature was 200 °C, with 70 V/150 μA ionization voltage/current. The mass spectrometer was set to scan m/z range 50 to 600, with ∼1 kV detector sensitivity. For analysis of pyruvate, the derivatization procedure was modified by the substitution of ethylhydroxylamine for methoxyamine, and the initial GC-MS oven temperature was 110 °C for 4 min, rose to 230 °C at 6 °C/min and then resuming the protocol described above. Metabolites were quantified against varied amounts of standard mixtures run in parallel and data were analyzed using Metaquant v1.3.5. Quantities were corrected for recovery using the *L*-norvaline internal standard. Following derivatization of G3P and DHAP, different isomers formed that were separately quantified. Though identical quantitation of both peaks is expected due to the same derivatization reaction being used on all standards and samples, this is not always the case, and thus average quantitation of both peaks were then used in each replicate (Table S5). These methods have been previously reported (92).

### PCA analysis

Dimensionality reduction method was carried out on all samples using Principal Component Analysis (PCA) in RStudio 2023.06.0 Build 421. Metabolites were read as.csv files and assigned as data matrices and PCA was carried out on sample groups using the *prcomp* function. Missing values were removed using *na.omit* and PCA plots were generated using ggplot2 (93).

### RRBS

100 to 200 mg of tumor tissue from 12 U87MG tumor xenografts (3 WT, 4 R132H, and 5 R132Q tumors, all randomly selected) and 9 HT1080∗ tumor xenografts (2 WT, 3 R132H with one failed data collection that was then eliminated, and 5 R132Q tumors, all randomly selected) was weighed and then shipped in dry ice for RRBS analysis at Active Motif (Carlsbad, CA). There, tissues were subjected to proteinase K digest solution (0.5% SDS, 0.5 mg/ml proteinase K, 100 mM EDTA, in TE pH 8) by allowing the samples to rotate overnight at 55 °C. For library preparation and sequencing, 100 ng of gDNA was digested with TaqaI at 65 °C for 2 h followed by MspI treatment at 37 °C overnight. Following enzymatic digestion, samples were used for library generation using the Ovation RRBS Methyl-Seq System (Tecan, Männedorf, Switzerland) following the manufacturer’s instructions. The digested DNA was randomly ligated, and, following fragment end repair, bisulfite was converted using the EpiTect Fast DNA Bisulfite Kit following the manufacturer’s protocol. After conversion and clean-up, samples were amplified, resuming the Ovation RRBS Methyl-Seq System protocol for library amplification and purification. Libraries were measured using the Agilent 2200 TapeStation System and quantified using the KAPA Library Quant Kit ABI Prism qPCR Mix. Libraries were sequenced on a NovaSeq 6000 (Illumina, San Diego, CA) at SE75 x 30M reads (single-end).

### RRBS data processing and analysis

Methylation calls were produced using Bismark and imported into the MethylKit R package (v. 1.28.0) (94). CpGs with more than ten reads were included in the downstream analysis. Filtered counts were normalized and CpG dinucleotides were merged (destrand = FALSE). Most variable CpGs across tumors were calculated as having a SD > 0.90. Samples were visualized using PCA and hierarchical clustering using the Ward method. For HT1080∗ tumors, to perform pairwise comparisons in methylation levels, samples of interest were merged into one object containing common CpGs. Pearson correlation was used to assess concordance between D2HG amount in the tumors and DNA methylation levels of CpG sites. Differential methylation analysis was performed using Fisher’s exact test and differentially methylated CpGs were selected based on q < 0.05 and percent methylation difference > 20%. For U87MG tumors, CpGs were first filtered to include the most variable sites (CpGs with SD > 10) and differential methylation analysis was performed as described, with a notable exception that CpGs were selected based on q < 0.05 and methylation difference > 10%. Feature annotation was done using ChIPseeker (v. 1.38.0) R package (95, 96). Genomic annotations for hg38 were retrieved using the TxDb.Hsapiens.UCSC.hg38.knownGene R package (97). GREAT analysis software tool (with default parameters) (98, 99) was applied to determine enriched GO terms near the differentially methylated CpGs. The entire filtered CpG sites used for differential methylation analysis was used as the genomic background for GREAT analysis.

### RNA-seq

RNA samples were processed at the University of California, San Diego Institute for Genomic Medicine (IGM). RNA samples were extracted from 12 U87MG tumor xenografts (3 WT, 4 R132H, and 5 R132Q tumors, all randomly selected and identical to those selected for RRBS analysis) and 10 HT1080∗ tumor xenografts (2 WT, 3 R132H, and 5 R132Q tumors, all randomly selected and identical to those selected for RRBS analysis) using QIAzol Lysis Reagent. Tumor tissue (100–200 mg) was placed in gentleMACS M tubes. 500 μl of QIAzol Lysis Reagent was added to the tissue, and the samples were dissociated mechanically using a gentleMACS Dissociator. All tubes were incubated at room temperature for 5 min, and cell lysate was transferred to an autoclaved Eppendorf tube. Chloroform (0.2 ml) was added, followed by 30 s vortexing and a 5 min incubation at room temperature. Following separation by centrifugation at 12,000 rpm for 15 min at 4 °C, the top of three total layers containing RNA was transferred to a new autoclaved Eppendorf tube. After adding 0.5 ml of isopropanol, samples were mixed by vortexing for 15 s and incubated at room temperature for 10 min. Samples were then clarified by centrifugation at 12,000 rpm for 10 min at 4 °C. The supernatant was aspirated and 1 ml of 75% ethanol was added to the pellet that was mixed using vortexing for 15 to 30 s. Samples were then separated by centrifugation at 7500 rpm for 5 min at 4 °C. The supernatant was removed, and the RNA pellets were air-dried for 5 min and then dissolved in 50 to 70 μl of RNase-free water. The integrity of RNA was assessed on a 1% agarose gel in 1x TAE buffer (0.4 M tris acetate pH 8.3 at room temperature, 0.01 M EDTA). An RNA quality control check was conducted at IGM and only high-quality RNA samples with RIN ≥ 7.4 were used to generate the RNA-seq libraries. Libraries were generated using the Illumina Ribo-Zero Plus rRNA Depletion Kit with IDT for Illumina RNA UD Indexes (Illumina, San Diego, CA). All samples were processed according to the manufacturer’s instructions. Generated libraries were multiplexed and sequenced with 150 base pair (bp) Paired End (PE150) to a depth of approximately 25 million reads per sample on an Illumina NovaSeq 6000. Samples were finally demultiplexed using bcl2fastq v2.20 Conversion Software (Illumina, San Diego, CA).

### RNA-seq data processing and analysis

The quality of the raw RNAseq data was evaluated using FASTQC v0.12.1 http://www.bioinformatics.babraham.ac.uk/projects/fastqc/. Based on the FASTQC reports, potential adapter contamination and low-quality sequences were identified and trimmed using Cutdapt v4.8 (100). Cutadapt parameters were set for paired-end reads, with a quality cutoff of 20 and a minimum filter length of 20. The genome index was constructed using the hg38 reference genome assembly obtained from the National Center for Biotechnology Information (NCBI) with the Spliced Transcripts Alignment to a Reference (STAR) aligner version v2.7.11b (101). Subsequently, quality-filtered reads were aligned to this genome index using STAR. The aligned reads were sorted by read name using sambamba v1.01 to prepare them for counting (102). featureCounts v2.0.6 was utilized to count the number of reads per annotated gene (103). The raw gene counts were normalized using DESeq2 to ensure differences in sample read counts represented biological differences rather than technical variation (104). DESeq2 was also used to identify differentially expressed genes (DEGs) in the mutants when compared to the WT form. Significant DEGs were defined as genes exhibiting an adjusted *p*-value (FDR) below 0.05. Pathway analysis was performed to identify significantly enriched gene sets from the Kyoto Encyclopedia of Genes and Genomes (KEGG), as well as the Gene Ontology (GO) databases covering biological processes, molecular functions, and cellular components using ClusterProfiler v4.10.1 (105, 106, 107). Significantly enriched, upregulated, and downregulated pathway alterations between sample groups were determined by an adjusted *p*-value (FDR) below 0.05 (see Tables S6 and S7).

### Statistical analysis

Statistics were completed as described here or as otherwise noted in the figure legends and were generated using Prism 10.3.10. Results are presented as mean ± SD. Significance was calculated using an unpaired *t* test for comparisons of two means or ANOVA for comparisons of three or more means and a Tukey *post hoc* test to identify differences between groups. Differences between means are considered statistically significant at the 95% level (*p* < 0.05). No stars or a label of ns is not significant unless otherwise indicated (see Fig. 1*A* for details).

### Scanning electron microscopy (SEM)

Cells were seeded at a density of 5 × 10^5^ (U87MG) or 2.5 × 10^5^ (HT1080∗) in a 10 cm cell culture treated dish and grown in high-glucose DMEM supplemented with 10% FBS. Cells were cultured for approximately 72 h at 37 °C with 5% CO_2_, and growth media was changed at approximately 36 h. After 72 h incubation, cell supernatant was aspirated off without disturbing the monolayer, and washed with DPBS (Gibco, Grand Island, NY) to remove residual FBS. Karnovsky's Fixative (1% glutaraldehyde, 4% paraformaldehyde in 0.1 M cacodylate buffer at pH 7.4) was added to cover the cells. After 1 h incubation at room temperature, the cells were stored at 4 °C until processed for SEM. After washing with 0.1 M cacodylate buffer, cells were immersion postfixed in 1% OsO_4_ for 2 h, then washed and dehydrated with a standard ethanol series. Coverslips were chemically dried with HMDS overnight, mounted on Al stubs and coated with 6 nm Pt. They were imaged and viewed on a FEI Quanta 450 FEG SEM.

### Migration and proliferation assays

To assess migration using a scratch assays, cells were counted and seeded in poly-d-lysine-coated Corning 96-well tissue culture plates to a density that would generate 80% confluency after 24 h. After a 24 h incubation at 37 °C with 5% CO_2_, a scratch was made using a P-20 pipette tip. Cells were washed and complete media was replaced with serum-free DMEM and cells were returned to incubation. Cell imaging was performed at 0, 2, 4, 6, 12, and 24 h using a Zeiss Axio Observer 7 Axiocam 712 mono with Zen 3.8 Pro software. To assess proliferation, cells were seeding in three 96-well plates using 500, 1000, 1500, and 2000 cells to obtain an initial standard curve. Cells were incubated for 24 h at 37 °C with 5% CO_2_ in DMEM supplemented with 10% FBS before using CellTiter-Glo (Promega) according to manufacturer's protocol. Proliferation was monitored by luminescence at 0, 24, and 48 h using a CLARIOstar *Plus* plate reader (BMG Labtech, Cary, NC).

### ROS assay

A ROS-Glo H_2_O_2_ ROS assay kit was used according to manufacturer protocol to assess ROS levels in U87MG cells. Briefly, U87MG cells were seeded in a 96-well cell culture plate coated with poly-d-lysine and cultured for approximately 72 h at 37 °C with 5% CO_2_ in DMEM supplemented with 10% FBS. At the end of incubation, the medium was removed from all wells. 80 microliters of DMEM containing either 12.5 mM *N*-acetyl-cysteine (in triplicate), or 6.25 μM cumene hydroperoxide (in duplicate), and 20 μl of substrate dilution buffer containing 125 μM H_2_O_2_ substrate was added to appropriate treatment wells for final treatment concentrations of 10 mM *N*-acetyl-cysteine, 5 μM of cumene hydroperoxide, and 25 μM H_2_O_2_ substrate. The plate was then incubated for 2.5 h at 37 °C with 5% CO_2_. Luciferin detection reagent was prepared by reconstituting lyophilized luciferin detection reagent with the supplied reconstitution buffer supplied by the assay kit. Immediately prior to use, 10 μl of *D*-cysteine and 10 μl of Signal Enhancer solution were added per 1 ml of luciferin detection reagent to produce ROS-Glo detection solution. After incubation, 100 μl of ROS-Glo Detection solution was added to each well. The plate was incubated for 20 min at room temperature, and luminescence was read with a CLARIOstar *Plus* plate reader.

### Public database analysis

The expression of TP53I3, MYC, and IL6 were compared in IDH1 mutant *versus* WT patient samples from Brain Lower Grade Glioma in the Cancer Genome Atlas (TCGA). Z-scores from all samples were associated with IDH mutational status and survival outcomes by accessing data on the Cancer Genomics Portal Website (13, 108, 109) and reconstructing in excel by unique patient ID. Of the 514 total cases, 415 were IDH mutant and 99 were IDH WT. Plots were generated using KMPlotter (110).

## Data availability

The complete set of RNAseq and RRBS data can be acquired *via* NCBI's Gene Expression Omnibus (GEO) under accession numbers GSE276109 (RNAseq) and GSE276110 (RRBS).

## Supporting information

This article contains supporting information.

## Conflict of interest

The authors declare that they have no conflicts of interest with the contents of this article.

## Supporting information

Supporting information

Supplemental TableS5

Supplemental TableS6

Supplemental TableS7
