# Supplementary material for: Catalytically distinct metabolic enzyme isocitrate dehydrogenase 1 mutants tune phenotype severity in tumor models
Source: J Biol Chem. 2025 Apr 4;301(5):108477. doi: 10.1016/j.jbc.2025.108477 (PMC12147185; doi:10.1016/j.jbc.2025.108477)

SUPPORTING INFORMATION

Catalytically distinct metabolic enzyme isocitrate dehydrogenase 1 mutants tune phenotype severity in tumor models

Ashley V. Schwartz, Grace Chao, Mikella Robinson, Brittany M. Conley, Mowaffaq Adam Ahmed Adam, Grace A. Wells, An Hoang, Elene Albekioni, Cecilia Gallo, Joi Weeks, Katelyn Yunker, Giovanni Quichocho, Uduak Z. George, Ingrid Niesman, Carrie D. House, Şevin Turcan, and Christal D. Sohl

Fig. S1. Steady-state kinetic analysis for WT and R132H and R132Q mutant mixtures.
Fig. S2. Western immunoblot analysis of exogenously expressed HA-tagged IDH1.
Fig. S3. Features of U87MG mouse xenografts.
Fig. S4. Features of HT1080* mouse xenografts.
Fig. S5. Western immunoblot analysis of HT1080* and U87MG xenografts.
Fig. S6. Correlation of D2HG levels.
Fig. S7. Distribution of differentially methylated CpG sites across chromosomes.
Fig. S8. Distribution of differentially methylated CpG sites by CpG island annotations.
Fig. S9. Transcriptome analysis using RNAseq of HT1080* xenograft tumors comparing IDH1 R132Q and IDH1 WT.
Fig. S10. Comparison of select metabolite levels across HT1080* and U87MG cells and xenografts.
Fig. S11. Transcriptome analysis using RNAseq of HT1080* xenograft tumors comparing IDH1 R132H and IDH1 WT.
Fig. S12. Transcriptome analysis using RNAseq of U87MG xenograft tumors comparing IDH1 R132Q and IDH1 WT.
Fig. S13. Transcriptome analysis using RNAseq of U87MG xenograft tumors comparing IDH1 R132H and IDH1 WT.
Fig. S14. Morphological, proliferation, and migration features of WT and mutant IDH1-expressing U87MG cells.
Fig. S15. Western immunoblot analysis assessing tumor-driving pathways in U87MG cell lines exogenously expressing HA-tagged IDH1.
Fig. S16. Low expression of TP53I3 and high expression of IL6 and MYC is associated with worse outcomes in gliomas with mutant IDH1 but not WT IDH1.
Fig. S17. ROS quantitation in U87MG cells in U87MG cell lines exogenously expressing HA-tagged IDH1.
Table S1. Features of HT1080* and U87MG tumor xenografts.

Table S2. Differentially expressed genes in pairwise comparisons of RNA-seq analysis.

Table S3. RNAseq transcript details for selected genes from HT1080* tumor xenografts.

Table S4. RNAseq transcript details for selected genes from U87MG tumor xenografts.

Table S5. Spreadsheet of metabolic panel of HT1080* and U87MG cell lines and tumors (separate file).

Table S6. Spreadsheet of RNAseq results of transcripts significantly altered in HT1080* xenografts expressing IDH1 R132Q versus R132H (separate file).

Table S7. Spreadsheet of RNAseq results of transcripts significantly altered in U87MG xenografts expressing IDH1 R132Q versus R132H (separate file).

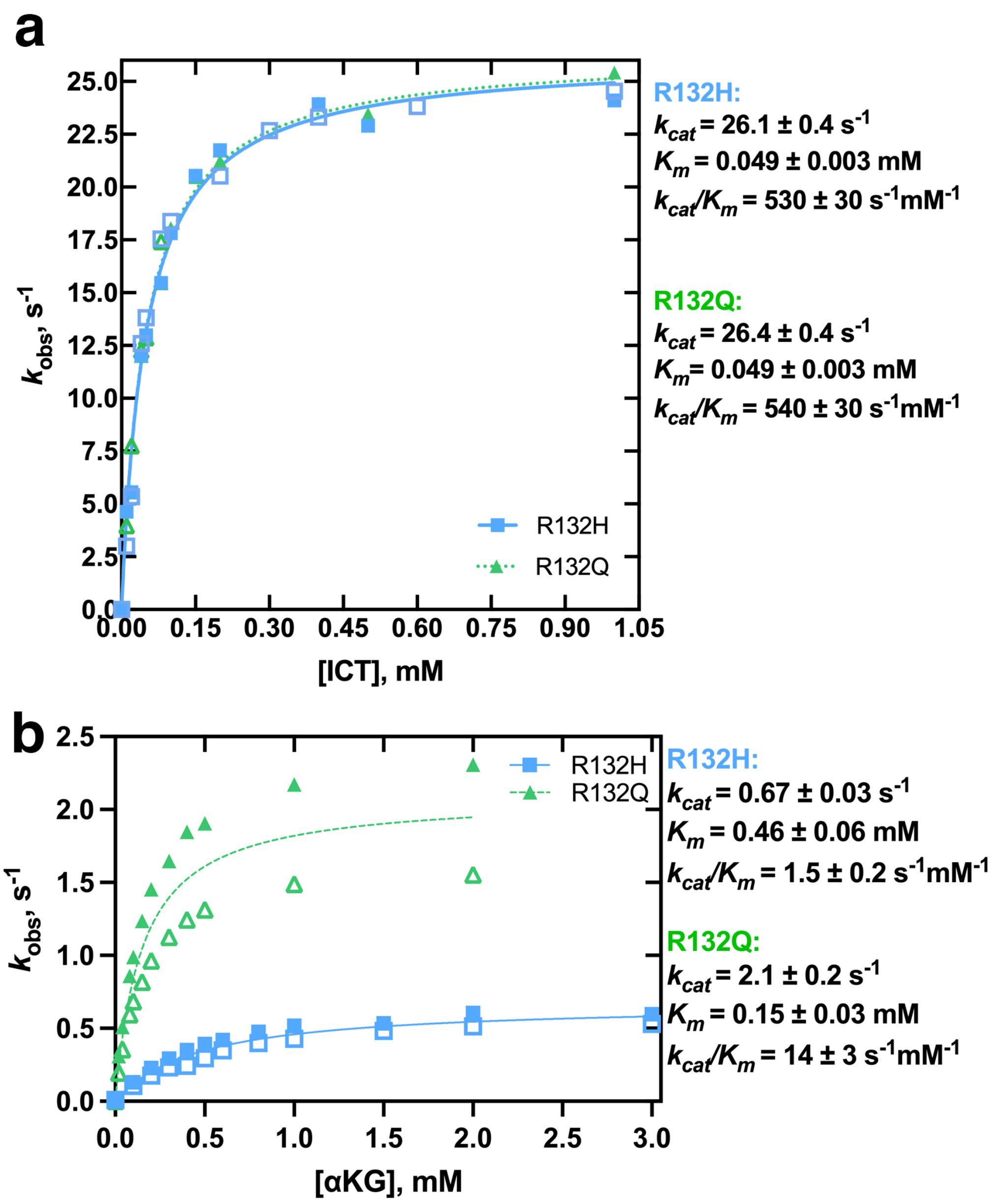

Fig. S1. Steady-state kinetic analysis for WT and R132H and R132Q mutant mixtures. Shown is a 1:1 mixture of WT and mutant IDH1 to allow heterodimerization to occur, though populations of WT:WT and mutant:mutant homodimers may also exist. **a**, Steady-state kinetic parameters for the conventional reaction of the conversion of ICT to αKG was measured as a function of varying substrate concentration. The following kinetic parameters were measured for a mixture of WT and R132H: $k_{cat,\ ICT \rightarrow \alpha KG}$ = 26.1 ± 0.4 s^{-1}; $K_{m,\ ICT}$ = 0.049 ± 0.003 mM; $k_{cat}/K_{m,\ ICT \rightarrow \alpha KG}$ = 530 ± 30 mM^{-1} s^{-1}. The following kinetic parameters were measured for a mixture of WT and R132Q: $k_{cat,\ ICT \rightarrow \alpha KG}$ = 26.4 ± 0.4 s^{-1}; $K_{m,\ ICT}$ = 0.049 ± 0.003 mM; $k_{cat}/K_{m,\ ICT \rightarrow \alpha KG}$ = 540 ± 30 mM^{-1} s^{-1}. **b**, Steady-state kinetic parameters for the neomorphic reaction of the conversion of αKG to D2HG were measured as a function of varying substrate concentration. The following kinetic parameters were measured for a mixture of WT and R132H: $k_{cat,\ \alpha KG \rightarrow D2HG}$ = 0.67 ± 0.03 s^{-1}; $K_{m,\ \alpha KG}$ = 0.46 ± 0.06 mM; $k_{cat}/K_{m,\ \alpha KG \rightarrow D2HG}$ = 1.5 ± 0.2 mM^{-1} s^{-1}. The following kinetic parameters were measured for a mixture of WT and R132Q: $k_{cat,\ \alpha KG \rightarrow D2HG}$ = 2.1 ± 0.2 s^{-1}; $K_{m,\ \alpha KG}$ = 0.15 ± 0.03 mM; $k_{cat}/K_{m,\ \alpha KG \rightarrow D2HG}$ = 14 ± 3 mM^{-1} s^{-1}. In all measurements, two biological replicates via two protein preparations are shown (open or closed symbols based on the protein preparation). Observed rate constants (k_{obs}) were calculated from the linear portion of plots of substrate concentration versus time. Kinetic parameters were calculated and reported as +/- SEM resulting from deviation of the mathematical fit.

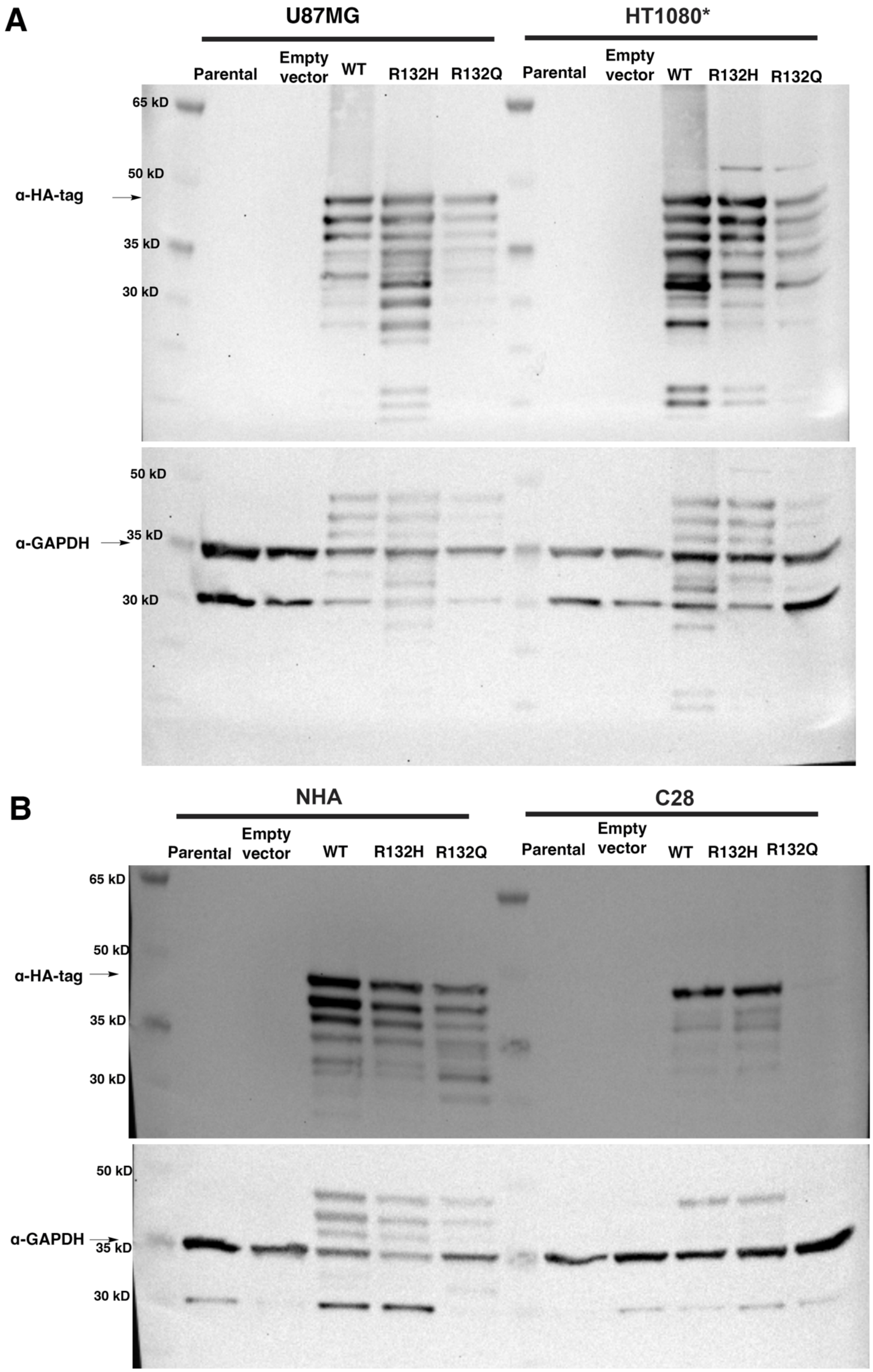

Fig. S2. Western immunoblot analysis of exogenously expressed HA-tagged IDH1. a, U87MG and HT1080* cell lines stably overexpressing HA-tagged WT or mutant IDH1. **b**, Normal human astrocytes (NHA) and C28 cell lines stably overexpressing HA-tagged WT or mutant IDH1.

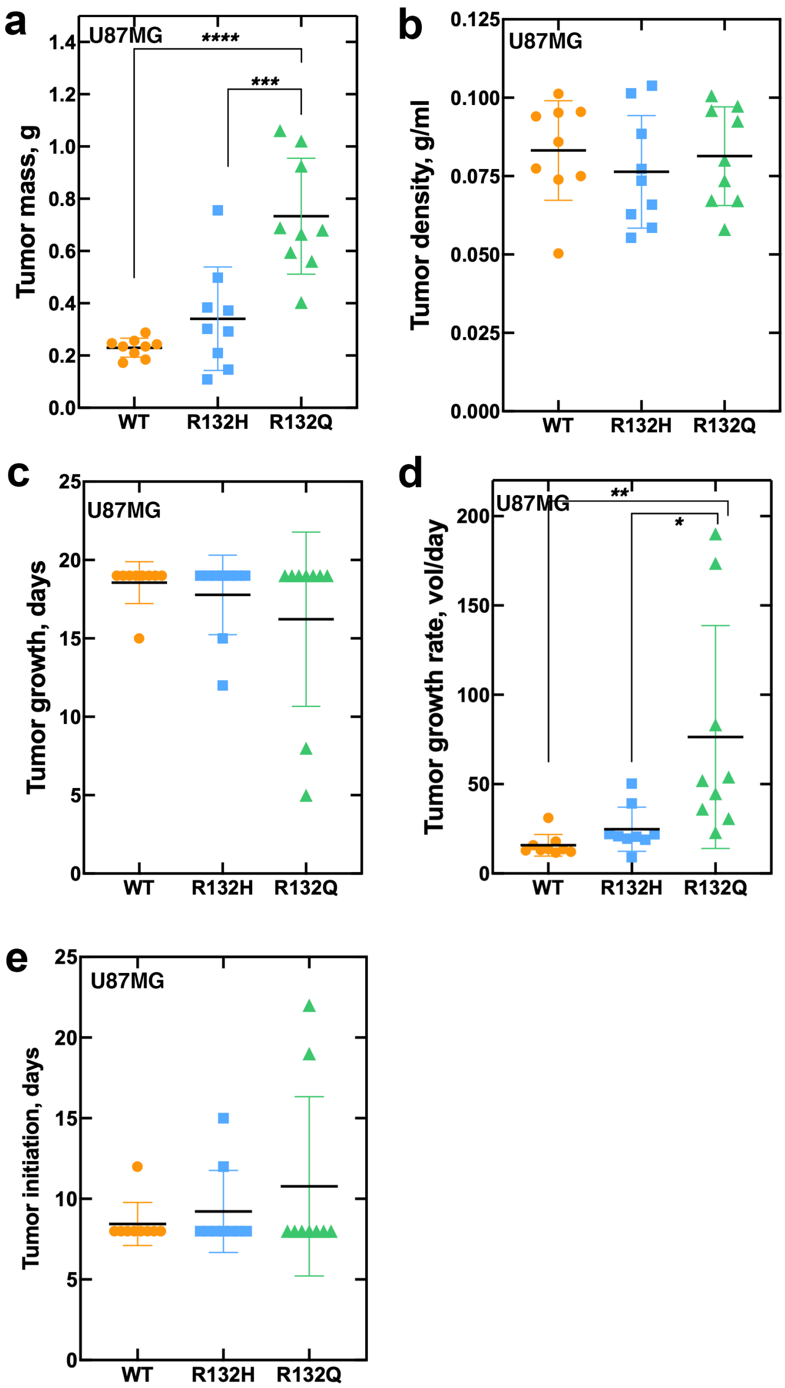

Fig. S3. Features of U87MG mouse xenografts. Nine biological mouse xenograft replicates were generated from each of the U87MG cell lines stably overexpressing IDH1 WT (orange circles), R132H (blue squares), or R132Q (green triangles). In all panels, the median is shown as a bar and error bars are shown as mean ± SD. Ordinary one-way ANOVA analyses, Tukey post-hoc tests were performed, and if a significant change was determined, the p value is indicated ($p \le 0.05$ (*), $p \le 0.01$ (**), $p \le 0.001$ (***), $p \le 0.0001$ (****)). **a**, The mass of all tumors formed were weighed at the day of euthanasia. **b**, The density of each tumor was calculated as mass (in g) per volume density. **c**, Tumor growth days were calculated by determining the number of days from tumor injection day to euthanasia date, and subtracting the number of days it took for the tumor to initiate from this number. **d**, The tumor growth rate was calculated by dividing the number of tumor growth days by the tumor volume in mL using a 1 mm^3 = 0.01 mL conversion. **e**, Tumor initiation was determined by the number of days for a tumor to form after injection.

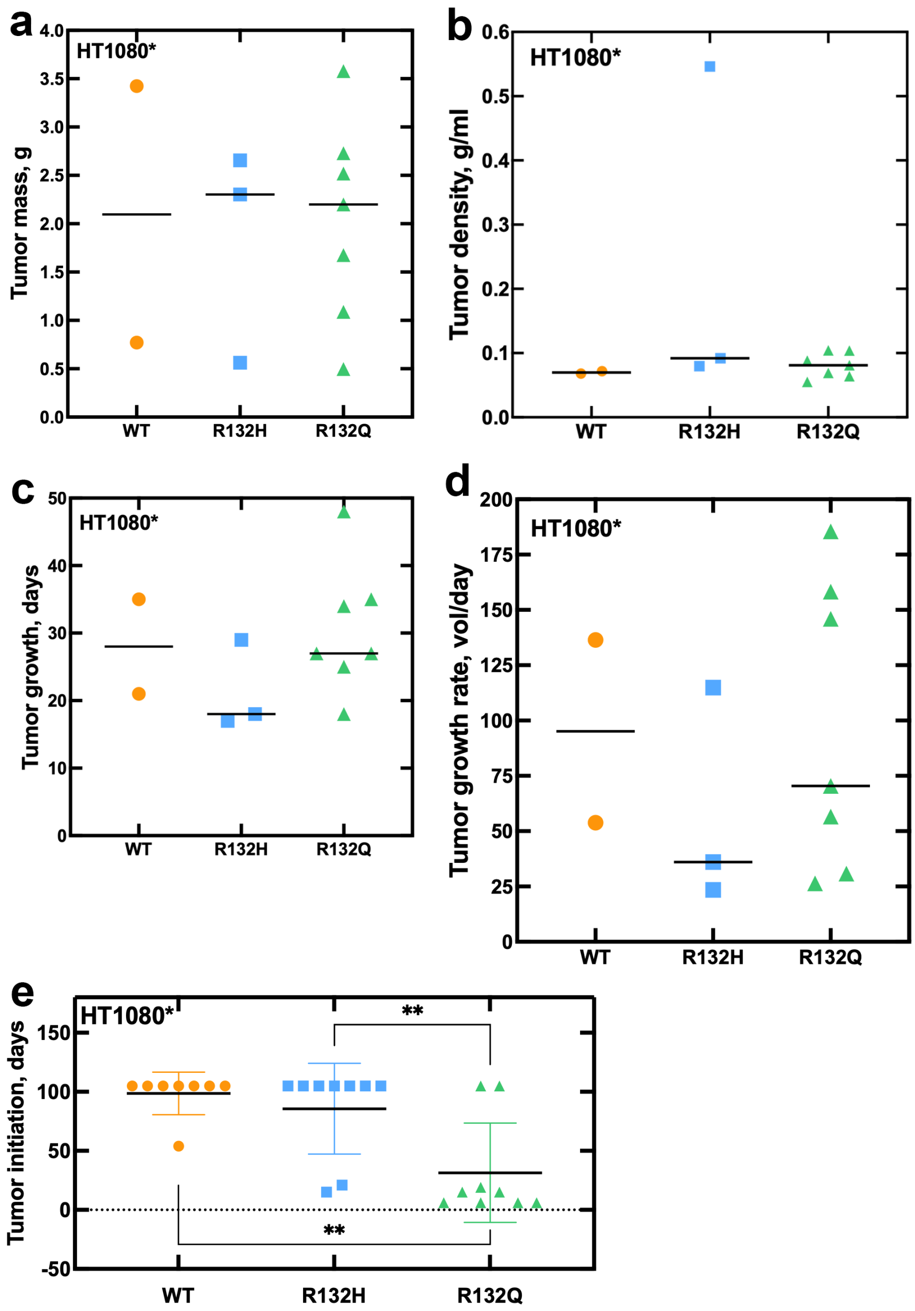

Fig. S4. Features of HT1080* mouse xenografts. Nine biological mouse xenograft replicates were generated from each of the HT1080* cell lines stably overexpressing IDH1 WT (orange circles), R132H (blue squares), or R132Q (green triangles). However, not all biological replicates generated a tumor. In **a-d**, only biological replicates where tumors formed were analyzed. In **e**, biological replicates without tumors forming by day 105 are also included. In all panels, the median is shown as a bar. Ordinary one-way ANOVA analyses, Tukey post-hoc tests were performed, and if a significant change was determined, the p value is indicated ($p \leq 0.01$ (**)). **a**, The mass of all tumors formed were weighed at the day of euthanasia. **b**, The density of each tumor was calculated as mass (in g) per volume density. **c**, Tumor growth days were calculated by determining the number of days from tumor injection day to euthanasia date, and subtracting the number of days it took for the tumor to initiate from this number. **d**, The tumor growth rate was calculated by dividing the number of tumor growth days by the tumor volume in mL using a 1 mm^3 = 0.01 mL conversion. **e**, Tumor initiation was determined by the number of days for a tumor to form after injection. Here error bars are shown as mean ± standard deviation (S.D.).

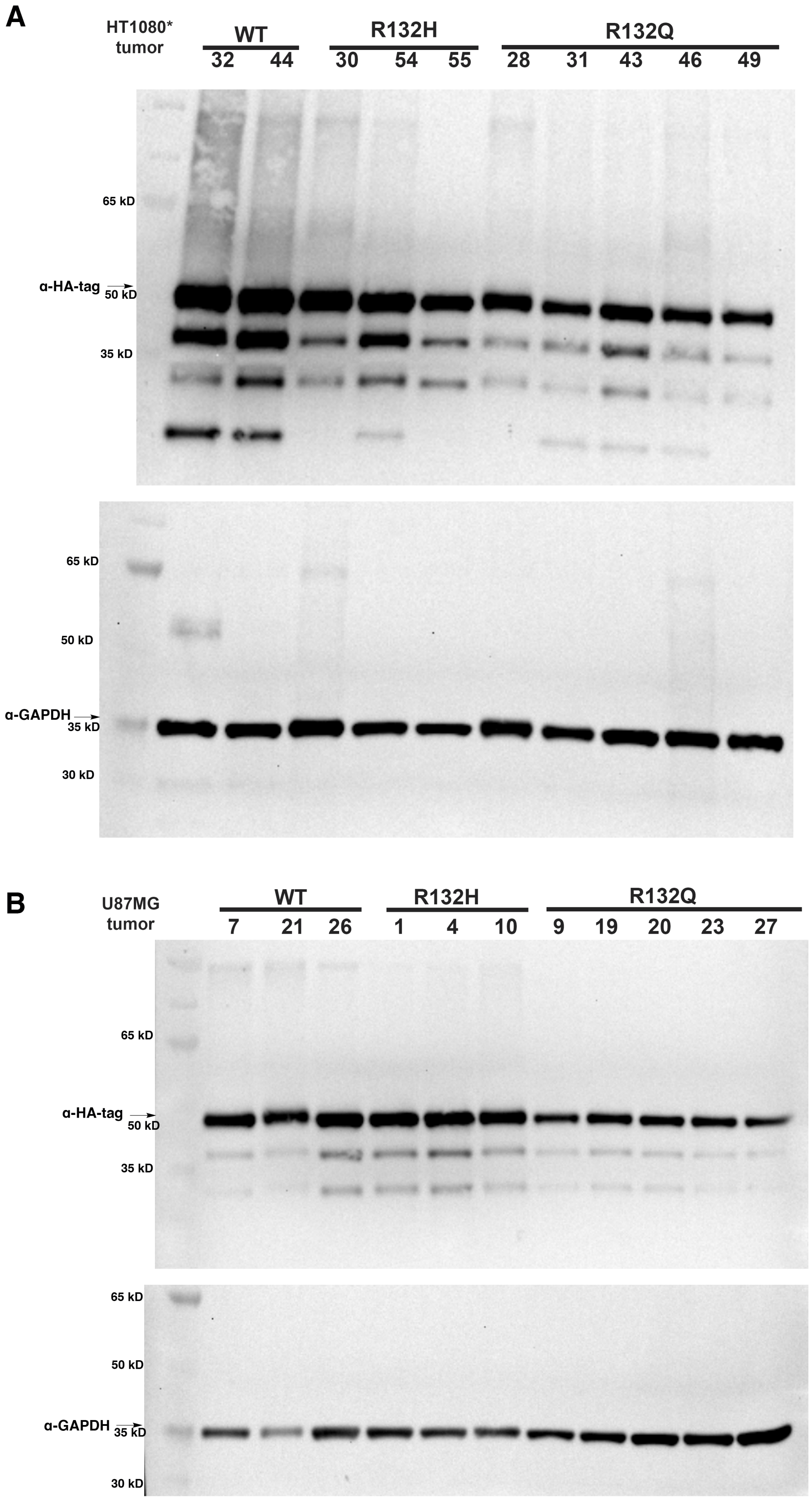

Fig. S5. Western immunoblot analysis of HT1080* and U87MG xenografts. a, Western immunoblot analysis of HT1080* xenograft tumors expressing HA-tagged WT and mutant IDH1. **b**, Western immunoblot analysis of U87MG xenograft tumors expressing HA-tagged WT and mutant IDH1.

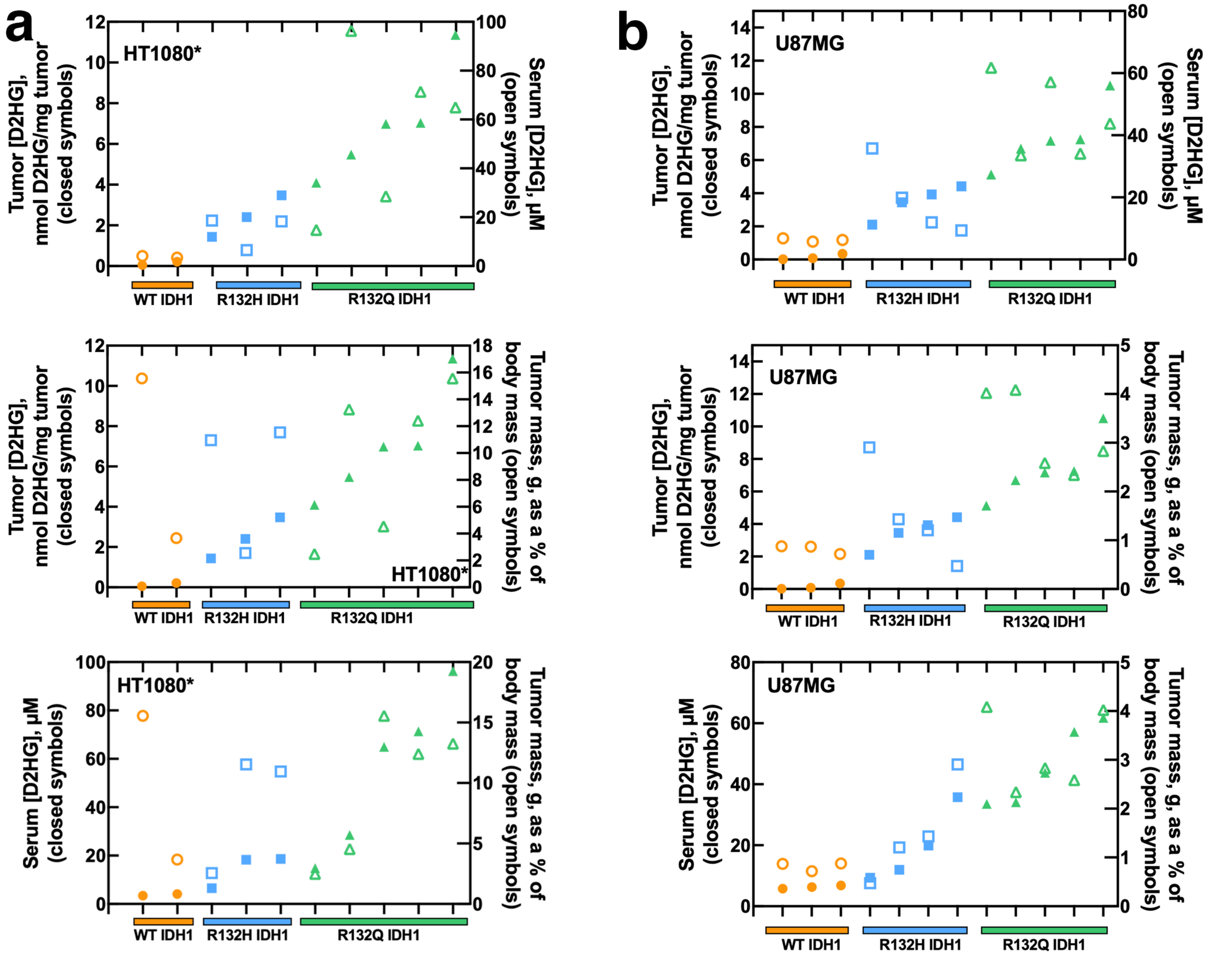

Fig. S6. Correlation of D2HG levels. a, For each of the HT1080* xenograft tumors (2 biological replicates for WT, 3 biological replicates for R132H, and 5 biological replicates for R132Q), D2HG concentrations in tumors are plotted versus D2HG concentrations in serum (top) and versus tumor mass as a percent of body mass (middle). Serum D2HG concentrations are plotted versus tumor mass as a percent of body mass (bottom). **b,** For U87MG xenografts, D2HG concentrations in tumors are plotted versus D2HG concentrations in serum (top) and versus tumor mass as a percent of body mass (middle). Serum D2HG concentrations are plotted versus tumor mass as a percent of body mass (bottom). Each point represents a single biological replicate.

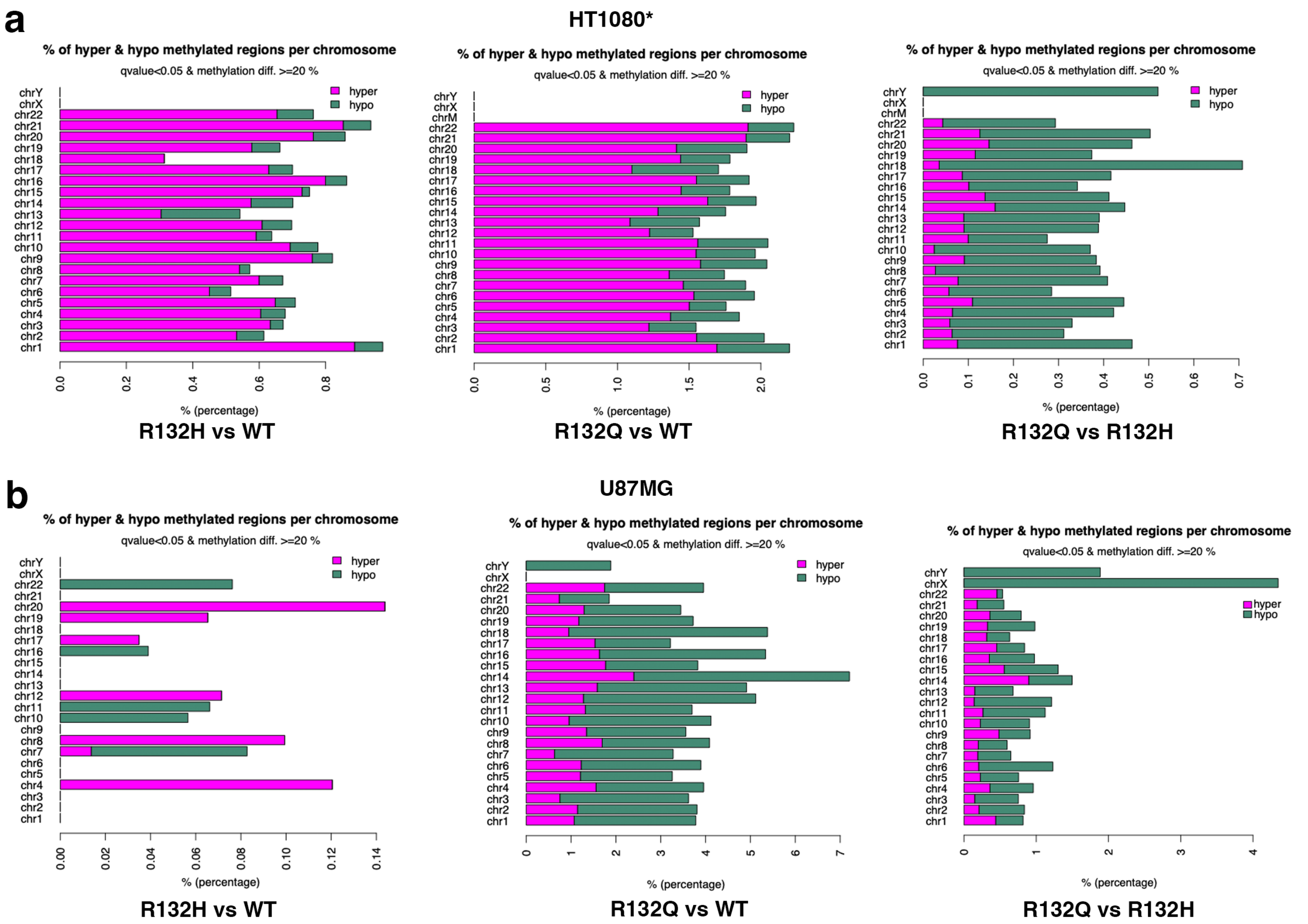

Fig. S7. Distribution of differentially methylated CpG sites across chromosomes. The distribution of differentially methylated CpG sites (DMS, % methylation difference ≥ 20 and q-value < 0.05) per chromosome in **a**, HT1080* and **b**, U87MG tumor xenograft RRBS data. Left panels, DMS in IDH1 R132H compared to IDH1 WT; middle panels, DMS in IDH1 R132Q compared to IDH1 WT, right panels, DMS in IDH1 R132Q compared to IDH1 R132H tumor samples. Hypermethylated (hyper) DMS are shown in pink and hypomethylated (hypo) DMS are shown in green. Two of two, three of three, and five of seven (randomized selection) of the xenograft tumors that formed HT1080* xenograft tumors upon IDH1 WT, R132H, or R132Q expression, respectively, were evaluated as biological replicates by RRBS, though one of the R132H xenograft tumors failed analysis. Three of nine, four of nine, and five of nine (randomized selection for each) of the xenograft tumors that formed U87MG xenograft tumors upon IDH1 WT, R132H, or R132Q expression, respectively, were evaluated as biological replicates by RRBS.

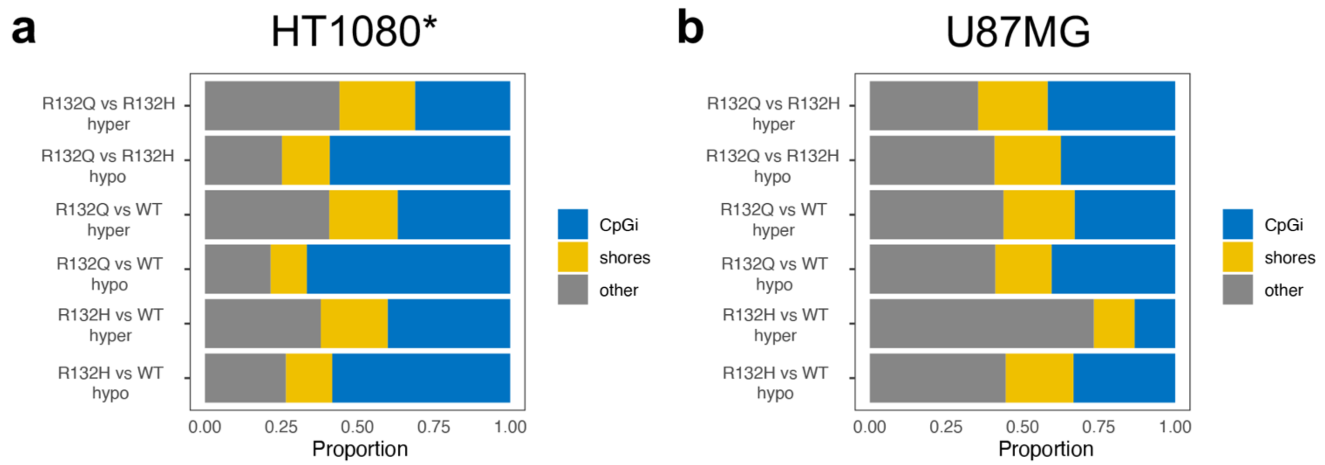

Fig. S8. Distribution of differentially methylated CpG sites by CpG island annotations. Bar plots showing the proportion of the DMS by CpG island annotations in **a**, HT1080*, and **b**, U87MG tumor xenografts. Blue indicates CpG islands (CpGi), yellow indicates shores, and gray indicates other.

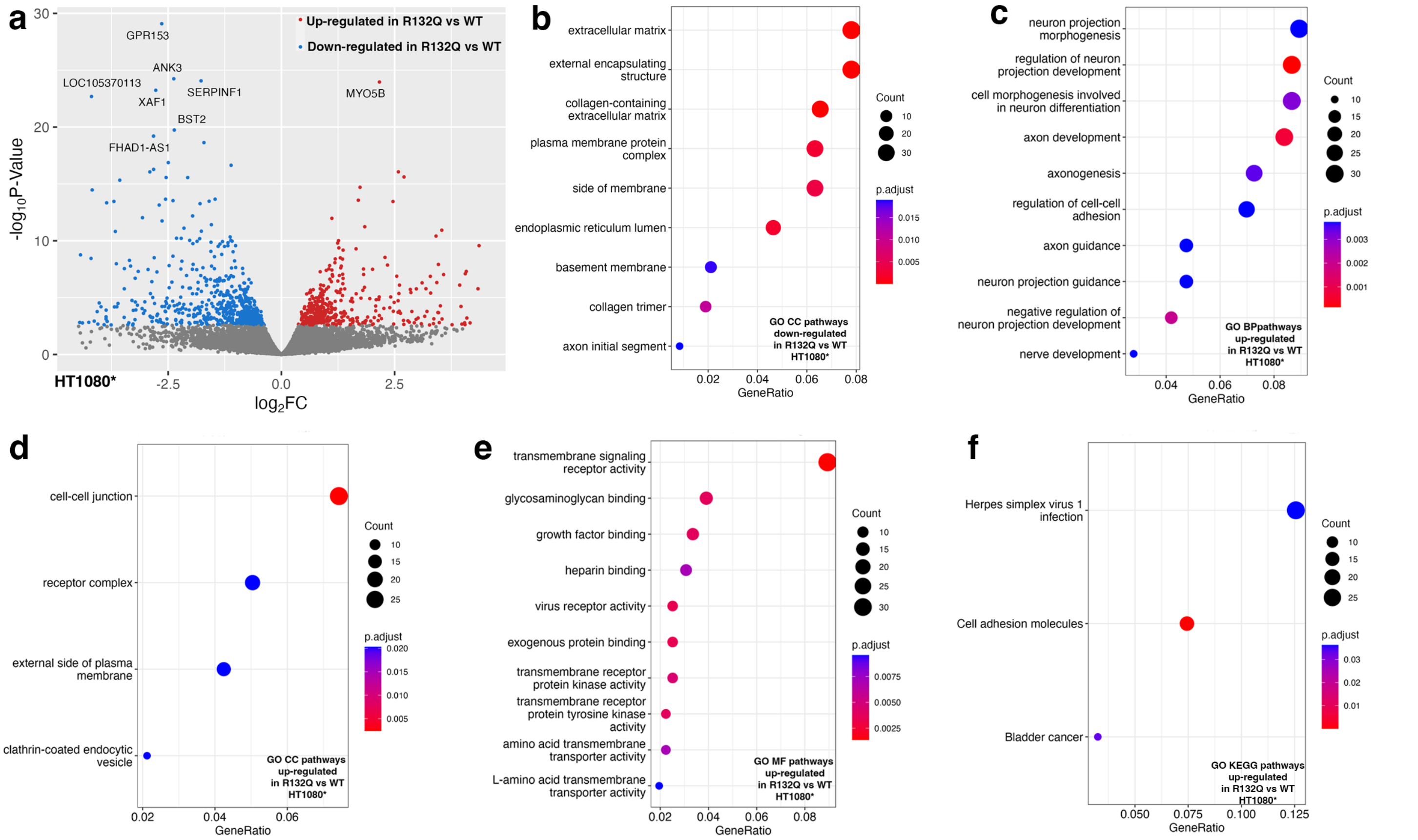

Fig. S9. Transcriptome analysis using RNAseq of HT1080* xenograft tumors comparing IDH1 R132Q and IDH1 WT. Two of two, three of three, and five of seven (randomized selection) of the xenograft tumors that formed HT1080* xenograft tumors upon IDH1 WT, R132H, or R132Q expression, respectively, were evaluated as biological replicates by RNAseq. Down-regulated pathways refer to categories where most genes show decreased expression levels in mutant versus WT IDH1. Up-regulated pathways refer to categories where most genes show increased expression levels in mutant versus WT IDH1. Number of genes is indicated in the count, with the p_{adjusted} value indicated by color. **a**, Volcano plot of differentially expressed transcripts comparing expression of IDH1 R132Q versus IDH1 WT. **b**, Cellular component (CC) pathways down-regulated in IDH1 R132Q versus IDH1 WT. **c**, Biological pathways (BP) up-regulated in IDH1 R132Q versus IDH1 WT. **d**, Cellular component (CC) pathways up-regulated in IDH1 R132Q versus IDH1 WT. **e**, Molecular function (MF) pathways up-regulated in IDH1 R132Q versus IDH1 WT. **f**, KEGG pathways up-regulated in IDH1 R132Q versus IDH1 WT.

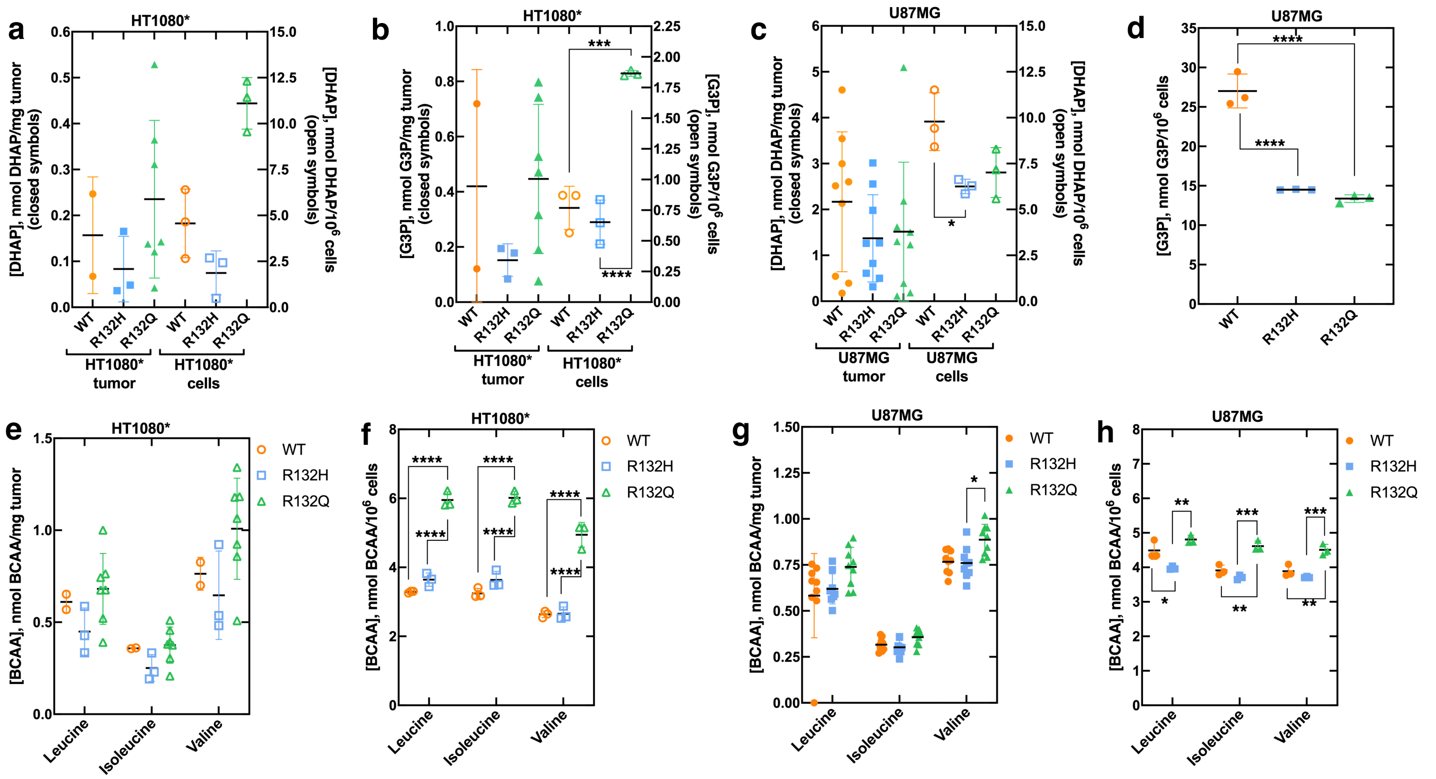

Fig. S10. Comparison of select metabolite levels across HT1080* and U87MG cells and xenografts. Nine biological mouse xenograft replicates were generated from each of the HT1080* cell lines stably overexpressing IDH1 WT (orange circles), R132H (blue squares), or R132Q (green triangles). However, not all biological replicates generated a tumor. For cell lines, three technical replicates are shown with the same color scheme used. For both tumors and cell lines, each point indicates a single replicate. **a**, Dihydroxyacetone phosphate (DHAP) concentrations in HT1080* tumor xenografts (left) and cell lines (right). **b**, Glyceraldehyde 3-phosphate (G3P) concentrations in HT1080* tumor xenografts (left) and cell lines (right). **c**, DHAP concentrations in U87MG tumor xenografts (left) and cell lines (right). **d**, G3P concentrations in U98MG cell lines (G3P was not = found at detectable levels in tumor samples). **e**, Concentrations of the branched-chain amino acids (BCAAs) leucine, isoleucine, and valine in HT1080* tumor xenografts. **f**, Concentrations of the BCAAs leucine, isoleucine, and valine in HT1080* cell lines. **g**, Concentrations of the BCAAs leucine, isoleucine, and valine in U87MG tumor xenografts. **h**, Concentrations of the BCAAs leucine, isoleucine, and valine in U87MG cell lines. In all panels, p values were determined by ordinary one-way ANOVA with **** $p \leq 0.0001$, *** $p \leq 0.001$, ** $p \leq 0.01$, * $p \leq 0.05$ for pairwise comparisons between IDH1 WT, R132H, and R132Q.

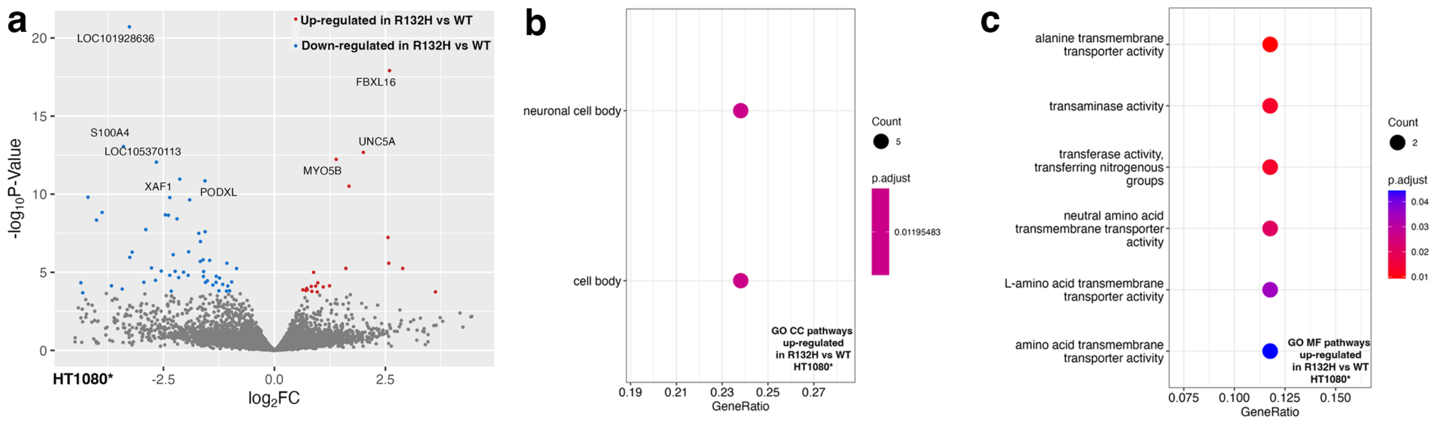

Fig. S11. Transcriptome analysis using RNAseq of HT1080* xenograft tumors comparing IDH1 R132H and IDH1 WT. Two of two, three of three, and five of seven (randomized selection) of the xenograft tumors that formed HT1080* xenograft tumors upon IDH1 WT, R132H, or R132Q expression, respectively, were evaluated as biological replicates by RNAseq. Down-regulated pathways refer to categories where most genes show decreased expression levels in mutant versus WT IDH1. Up-regulated pathways refer to categories where most genes show increased expression levels in mutant versus WT IDH1. Number of genes is indicated in the count, with the p_{adjusted} value indicated by color. **a**, Volcano plot of differentially expressed transcripts comparing expression of IDH1 R132H versus IDH1 WT. **b**, Cellular component (CC) pathways down-regulated in IDH1 R132H versus IDH1 WT. **c**, Molecular function (MF) pathways up-regulated in IDH1 R132H versus IDH1 WT.

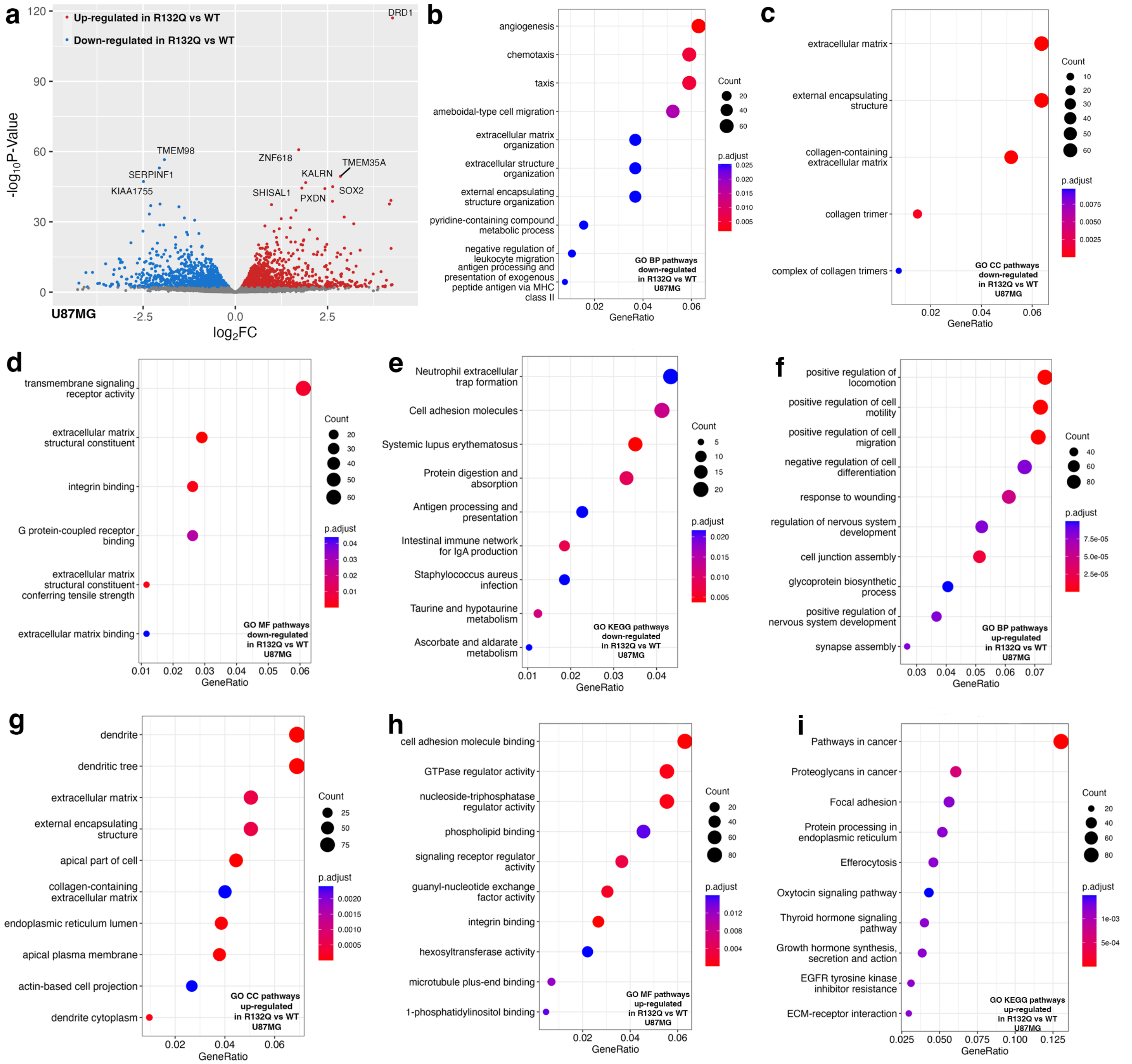

Fig. S12. Transcriptome analysis using RNAseq of U87MG xenograft tumors comparing IDH1 R132Q and IDH1 WT. Three of nine, four of nine, and five of nine (randomized selection for each) of the xenograft tumors that formed U87MG xenograft tumors upon IDH1 WT, R132H, or R132Q expression, respectively, were evaluated as biological replicates by RNAseq. Down-regulated pathways refer to categories where most genes show decreased expression levels in mutant versus WT IDH1. Up-regulated pathways refer to categories where most genes show increased expression levels in mutant versus WT IDH1. Number of genes is indicated in the count, with the p_{adjusted} value indicated by color. **a**, Volcano plot of differentially expressed transcripts comparing expression of IDH1 R132Q versus IDH1 WT. **b**, Biological pathways (BP) down-regulated in IDH1 R132Q versus IDH1 WT. **c**, Cellular component (CC) pathways down-regulated in IDH1 R132Q versus IDH1 WT. **d**, Molecular function (MF) pathways down-regulated in IDH1 R132Q versus IDH1 WT. **e**, KEGG pathways down-regulated in IDH1 R132Q versus IDH1 WT. **f**, BP up-regulated in IDH1 R132Q versus IDH1 WT. **g**, CC pathways up-regulated in IDH1 R132Q versus IDH1 WT. **h**, MF pathways up-regulated in IDH1 R132Q versus IDH1 WT. **i**, KEGG pathways up-regulated in IDH1 R132Q versus IDH1 WT.

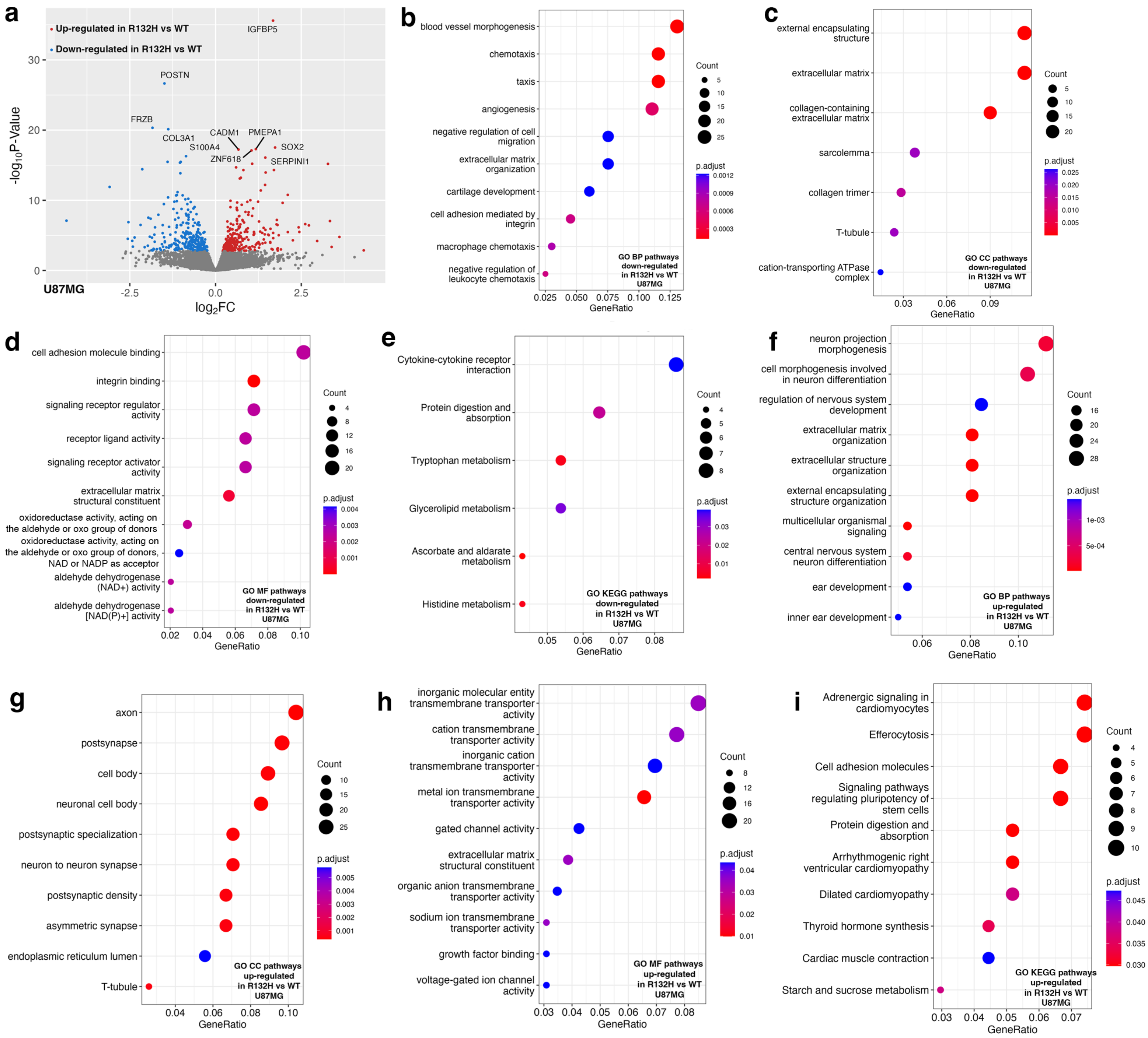

Fig. S13. Transcriptome analysis using RNAseq of U87MG xenograft tumors comparing IDH1 R132H and IDH1 WT. Three of nine, four of nine, and five of nine (randomized selection for each) of the xenograft tumors that formed U87MG xenograft tumors upon IDH1 WT, R132H, or R132Q expression, respectively, were evaluated as biological replicates by RNAseq. Down-regulated pathways refer to categories where most genes show decreased expression levels in mutant versus WT IDH1. Up-regulated pathways refer to categories where most genes show increased expression levels in mutant versus WT IDH1. Number of genes is indicated in the count, with the p_{adjusted} value indicated by color. **a**, Volcano plot of differentially expressed transcripts comparing expression of IDH1 R132H versus IDH1 WT. **b**, Biological pathways (BP) down-regulated in IDH1 R132H versus IDH1 WT. **c**, Cellular component (CC) pathways down-regulated in IDH1 R132H versus IDH1 WT. **d**, Molecular function (MF) pathways down-regulated in IDH1 R132H versus IDH1 WT. **e**, KEGG pathways down-regulated in IDH1 R132H versus IDH1 WT. **f**, BP up-regulated in IDH1 R132H versus IDH1 WT. **g**, CC pathways up-regulated in IDH1 R132H versus IDH1 WT. **h**, MF pathways up-regulated in IDH1 R132H versus IDH1 WT. **i**, KEGG pathways up-regulated in IDH1 R132H versus IDH1 WT.

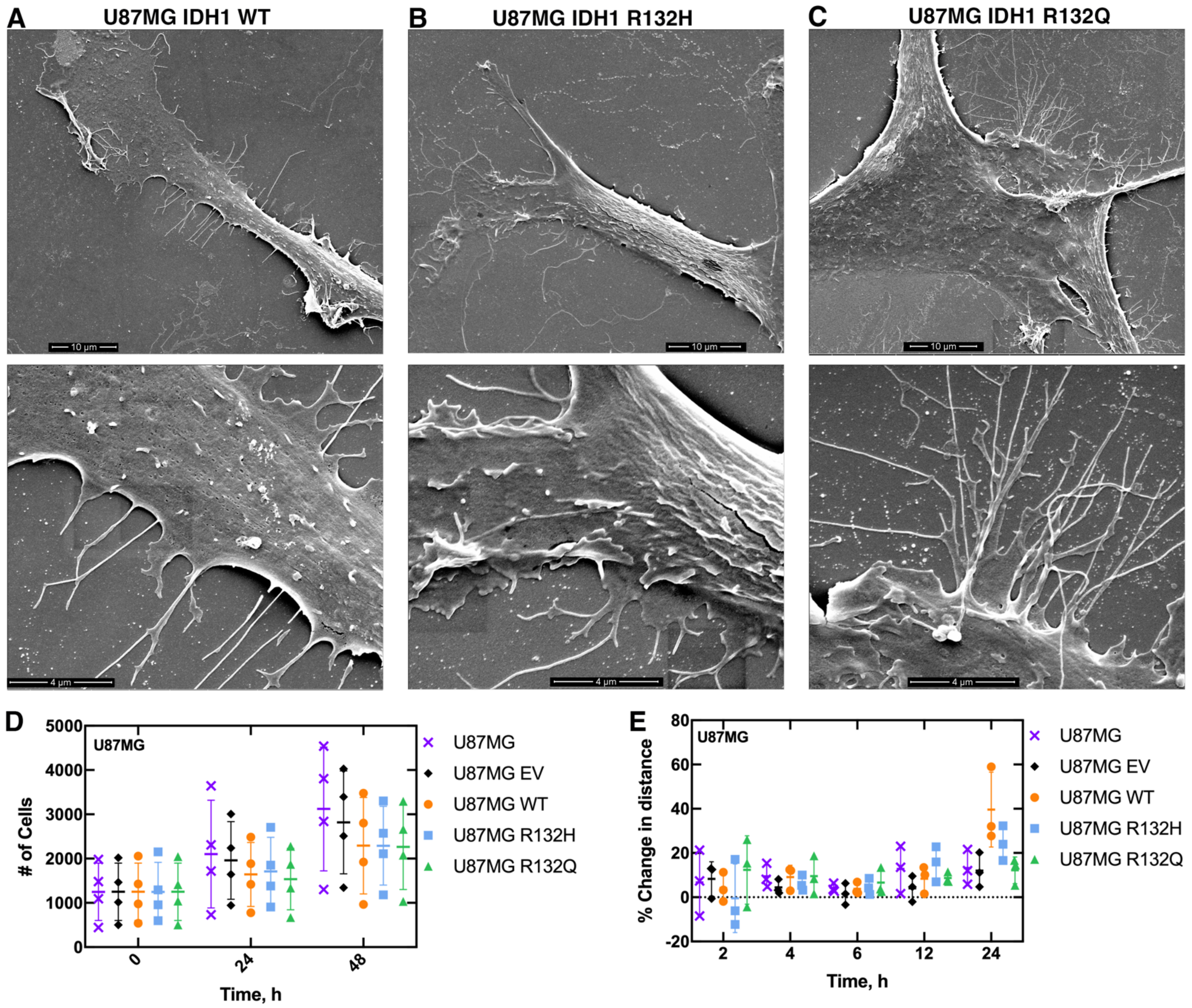

Fig. S14. Morphological, proliferation, and migration features of WT and mutant IDH1-expressing U87MG cells. a, Scanning electron (SE) micrograph of U87MG cells expressing HA-tagged IDH1 WT. **b**, SE micrograph of U87MG cells expressing HA-tagged IDH1 R132H. **c**, SE micrograph of U87MG cells expressing HA-tagged IDH1 R132Q. In **d** and **e**, U87MG cells are indicated as follows: parental (purple x, empty vector (EV) control (black diamonds), or stably expressing HA-tagged IDH1 WT (orange circles), R132H (blue squares), or R132Q (green triangles.) (parental, empty vector (EV), or stably overexpressing IDH1 WT, R132H, or R132Q).**d**, Proliferation assay of four technical replicates of U87MG cells. **e**, Wound healing (scratch) assay of three technical replicates of U87MG cells expressing HA-tagged IDH1 R132H.

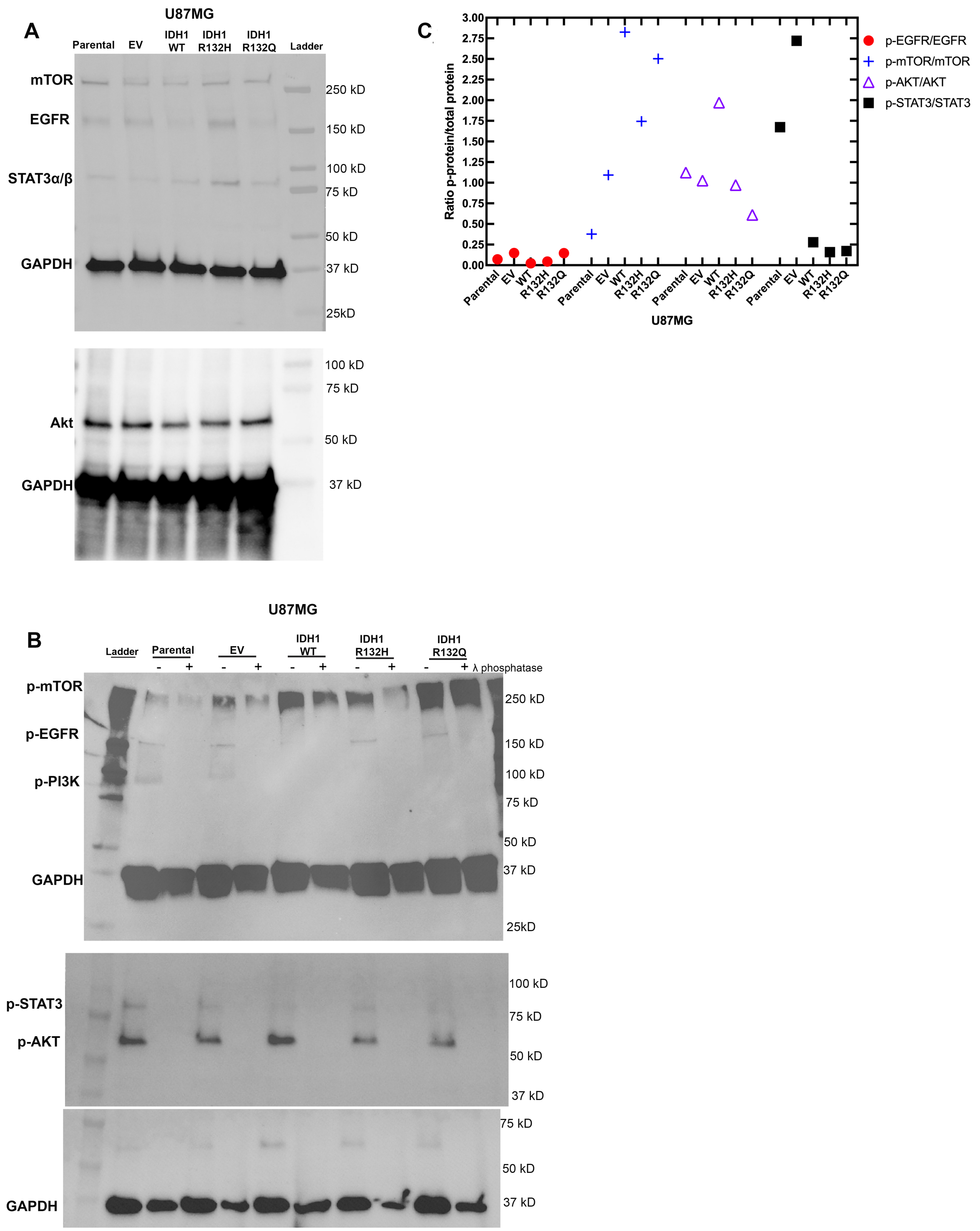

Fig. S15. Western immunoblot analysis assessing tumor-driving pathways in U87MG cell lines exogenously expressing HA-tagged IDH1. a, Shown are mTOR, EGFR, STAT3, and AKT levels in U87MG parental cells, or stably overexpressing empty vector (EV), IDH1 WT, IDH1 R132H, or IDH1 R132Q. **b**, Shown are phosphorylated mTOR (p-mTOR), phosphorylated EGFR (p-EGFR), phosphorylated PI3K (p-PI3K), phosphorylated STAT3 (p-STAT3), and phosphorylated AKT (pAKT) levels in U87MG parental cells, or stably overexpressing empty vector (EV), IDH1 WT, IDH1 R132H, or IDH1 R132Q. Lambda phosphatase treatment was used as a control. In **a** and **b**, GAPDH is shown as the loading control for each gel series, with loading also normalized to total protein levels. **c**, Intensity of phosphorylated protein divided by total protein is shown based on quantitation of the western immunoblots shown in panels **a** and **b**.

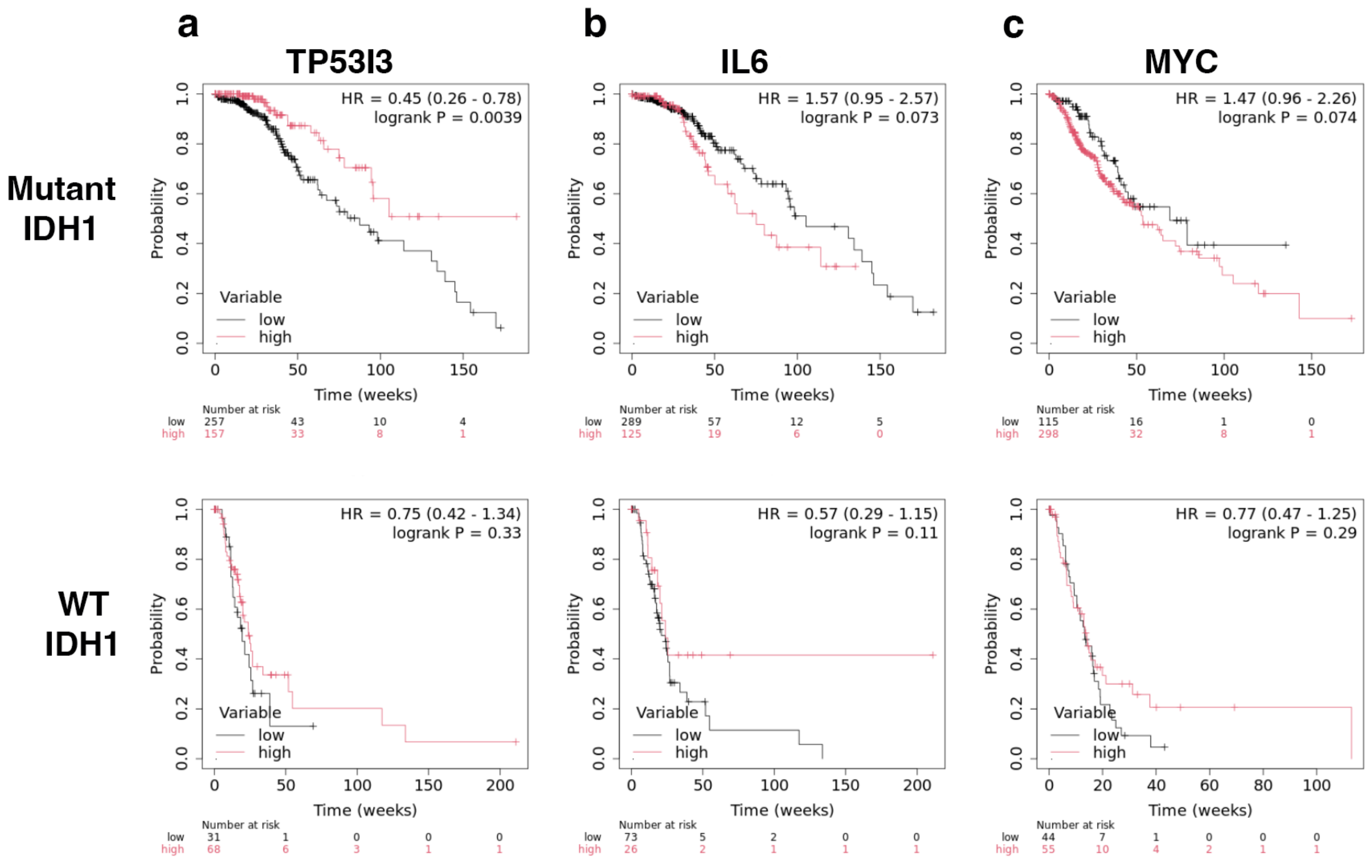

Fig. S16. Low expression of TP53I3 and high expression of IL6 and MYC is associated with worse outcomes in gliomas with mutant IDH1 but not WT IDH1. Kaplan-Meier plots comparing high expression (red) and low expression (black) of select genes in gliomas with mutant IDH1 (n=415) compared to WT IDH1 (n=99) collected from the Brain Lower Grade Glioma data set in TCGA. **a**, Expression of TP53I3 and overall survival of gliomas with mutant IDH1 compared to WT IDH1. **b**, Expression of IL6 and overall survival of gliomas with mutant IDH1 compared to WT IDH1. **c**, Expression of MYC and progression-free survival of gliomas with mutant IDH1 compared to WT IDH1.

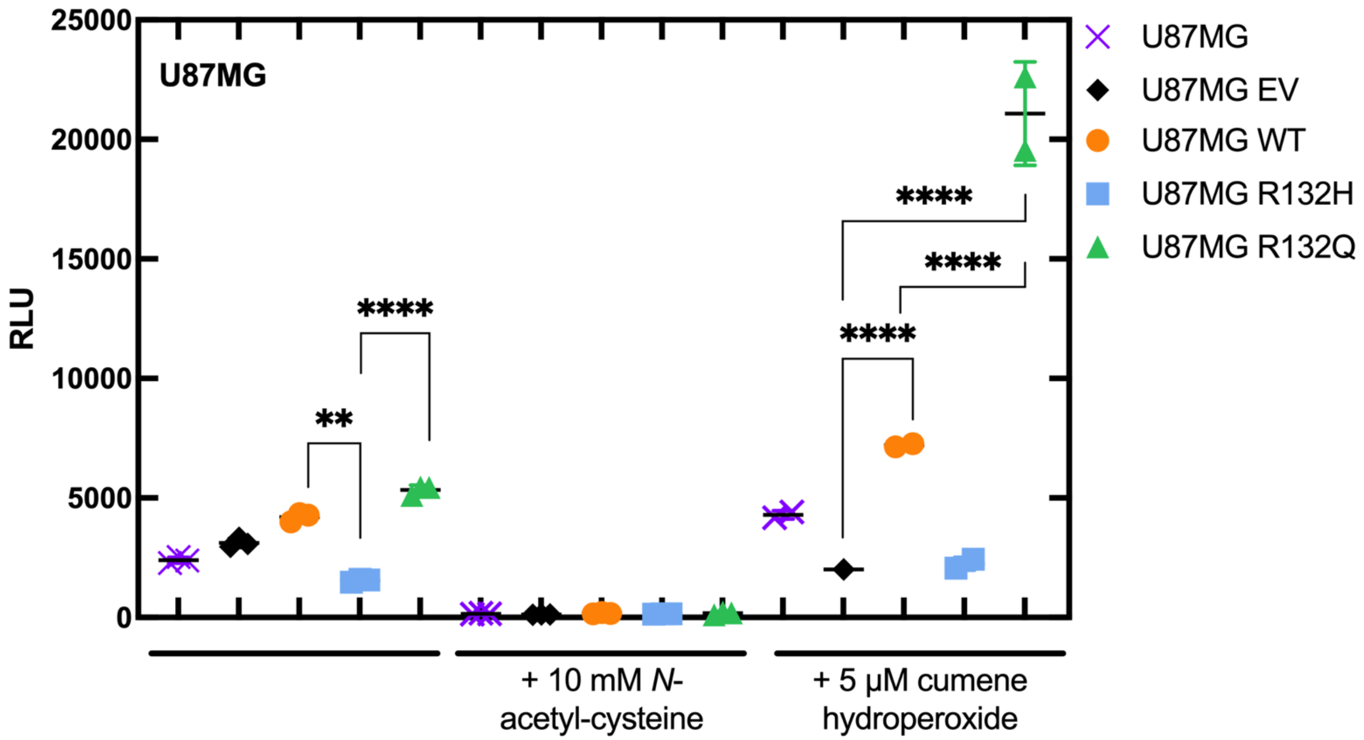

Fig. S17. ROS quantitation in U87MG cells in U87MG cell lines exogenously expressing HA-tagged IDH1. Shown are ROS in U87MG parental (purple x, empty vector (EV) control (black diamonds), or stably expressing HA-tagged IDH1 WT (orange circles), R132H (blue squares), or R132Q (green triangles.) (parental, empty vector (EV), or stably overexpressing IDH1 WT, R132H, or R132Q). Cells were either untreated (in triplicate), treated with the antioxidant *N*-acetyl-cysteine (10 mM, in triplicate) for 2.5 h, or treated with the ROS-inducing reagent cumene hydroperoxide (5 µM, in duplicate) for 2.5 h. Ordinary one-way ANOVA analyses, Tukey post-hoc tests were performed, and if a significant change was determined, the p value is indicated ($p \leq 0.05$ (*), $p \leq 0.01$ (**), $p \leq 0.001$ (***), $p \leq 0.0001$ (****)).

Table S1. Features of HT1080* and U87MG tumor xenografts.

Xenograft	IDH1 (ID #)	Tumor weight, g, as a % of body weight	[Tumor 2HG], nmol tumor D2HG/mg tumor	[Serum 2HG], µM	Number of covered CpGs (minCov > 10)	IDH1 transcripts (RNAseq)
HT1080*	WT (32)	3.67	0.21	4.12	1,688,753	26,331
HT1080*	WT (44)	15.56	0.057	3.46	2,098,845	41,577
HT1080*	R132H (30)	10.96	1.44	18.63	1,548,982	11,325
HT1080*	R132H (54)	11.55	3.48	18.33	2,102,976	28,180
HT1080*	R132H (55)	2.56	2.40	6.50	RRBS analysis failed	10,981
HT1080*	R132Q (28)	2.49	4.10	14.83	1,056,926	9,501
HT1080*	R132Q (31)	4.54	6.99	28.56	1,815,817	12,003
HT1080*	R132Q (34)	10.0	1.14	29.74	ND	ND
HT1080*	R132Q (43)	12.41	7.04	71.40	1,996,037	13,976
HT1080*	R132Q (46)	15.56	11.36	64.94	2,247,374	9,553
HT1080*	R132Q (49)	13.25	5.48	96.28	1,616,142	12,640
HT1080*	R132Q (52)	7.61	4.67	45.60	ND	ND
U87MG	WT (7)	0.88	0.021	6.81	1,686,541	18,566
U87MG	WT (18)	1.07	0.046	6.23	ND	ND
U87MG	WT (11)	1.07	0.064	5.31	ND	ND
U87MG	WT (13)	0.80	0.073	6.65	ND	ND
U87MG	WT (21)	0.87	0.075	5.76	1,670,198	18,608
U87MG	WT (17)	1.01	0.10	5.67	ND	ND
U87MG	WT (2)	1.07	0.14	10.20	ND	ND
U87MG	WT (26)	0.72	0.34	6.27	1,643,486	15,668
U87MG	WT (6)	1.25	0.55	6.19	ND	ND
U87MG	R132H (1)	2.91	2.11	35.78	1,889,953	20,244
U87MG	R132H (15)	1.74	2.77	17.55	ND	ND
U87MG	R132H (16)	0.91	3.29	9.54	ND	ND
U87MG	R132H (4)	1.43	3.45	19.89	1,794,986	16,962
U87MG	R132H (25)	0.63	3.47	11.60	ND	ND
U87MG	R132H (12)	2.26	3.48	15.83	RRBS analysis failed	ND
U87MG	R132H (22)	1.32	3.63	14.89	ND	ND
U87MG	R132H (10)	1.21	3.94	11.97	1,858,544	21,612
U87MG	R132H (24)	0.47	4.42	9.39	1,763,802	18,808
U87MG	R132Q (20)	4.02	5.13	61.76	1,395,526	12,249
U87MG	R132Q (3)	2.77	5.86	54.93	ND	ND
U87MG	R132Q (5)	4.42	6.04	42.28	ND	ND
U87MG	R132Q (14)	2.87	6.07	47.51	ND	ND
U87MG	R132Q (9)	4.08	6.70	33.58	1,841,212	12,188
U87MG	R132Q (27)	2.59	7.17	57.15	1,531,687	12,688
U87MG	R132Q (8)	1.75	7.22	34.46	ND	ND
U87MG	R132Q (19)	2.34	7.25	34.07	782,672	10,521
U87MG	R132Q (23)	2.83	10.51	43.77	1,567,435	11,596

Table S2. Differentially expressed genes in pairwise comparisons of RNA-seq analysis.

	R132H vs WT HT1080*	R132Q vs WT HT1080*	R132Q vs WT HT1080*	R132H vs WT U87MG	R132Q vs WT U87MG	R132H vs R132Q U87MG
Total number of genes	20002	20642	18643	18754	21076	17868
Total number of significant genes (padj < 0.05)	83	1062	959	573	2994	801
Total number of significant genes (padj < 0.05 and abs(log2FC) > 1)	71	592	327	153	837	173
Total number of significantly upregulated genes (padj < 0.05 and abs(log2FC) > 0)	21	470	518	307	1619	439
Total number of significantly upregulated genes (padj < 0.05 and abs(log2FC) > 1)	11	247	257	68	389	81
Total number of significantly downregulated genes (padj < 0.05 and abs(log2FC) > 0)	62	592	441	266	1375	362
Total number of significantly downregulated genes (padj < 0.05 and abs(log2FC) > 1)	60	345	70	85	448	92

Table S3. RNAseq transcript details for selected genes from HT1080* tumor xenografts. The relative change in Log2 FC is shown in a blue (relative decrease in transcripts) to red (relative increase of transcripts), and significantly altered genes based on p_{adj} values are notated in a white (non-significant) to grey (increasing significance) scale.

	R132Q vs R132H		R132Q vs WT		R132H vs WT	
GeneID	Log2 FC	padj	Log2 FC	padj	Log2 FC	padj
AFDN	0.52	0.00	0.68	0.00	0.15	1.00
ANXA6	-0.38	0.02	-0.48	0.03	-0.09	1.00
BDNF	-0.15	0.81	-0.16	0.85	-0.02	1.00
BMP7	2.64	0.07	2.22	0.34	NA	NA
CBL	0.45	0.02	0.46	0.25	0.01	1.00
CASP9	-0.65	0.00	-0.48	0.30	0.18	1.00
COL9A2	0.49	0.78	-2.52	0.04	-3.01	0.28
CCND2	3.83	0.04	2.22	0.39	NA	NA
CDK6	0.70	0.00	0.50	0.17	-0.19	1.00
EGFR	0.99	0.00	1.10	0.00	0.12	1.00
EPHB2	0.39	0.04	0.35	0.27	-0.04	1.00
ETS1	0.67	0.00	0.77	0.01	0.11	1.00
FZD1	-0.65	0.00	-0.72	0.11	-0.07	0.91
FZD9	NA	NA	-1.94	0.14	-2.50	0.71
JAG1	1.15	0.00	1.01	0.01	-0.14	1.00
KIRREL3	0.13	0.95	0.57	0.71	0.44	1.00
NKD1	-0.30	0.72	-1.41	0.00	-1.10	0.92
MYC	0.94	0.00	1.31	0.00	0.37	1.00
RALA	0.52	0.00	0.32	0.55	-0.20	1.00
RASAL2	0.72	0.00	0.72	0.00	0.00	1.00
SEMA3A	1.35	0.00	0.79	0.00	-0.55	1.00
TCF7	0.50	0.76	-0.59	0.80	-1.09	1.00
TP53	-0.12	0.68	-0.23	0.44	-0.10	1.00
TP53I3	-0.40	0.03	-0.79	0.00	-0.38	1.00
VEGFC	0.81	0.00	0.48	0.14	-0.32	1.00
WNT9A	-0.31	0.41	-1.07	0.00	-0.75	0.52
WNT10B	0.95	0.06	2.09	0.00	1.15	1.00

Log2 FC
1
0
-1

padj
0.05
0

Table S4. RNAseq transcript details for selected genes from U87MG tumor xenografts. The relative change in Log2 FC is shown in a blue (relative decrease in transcripts) to red (relative increase of transcripts), and significantly altered genes based on p_{adj} values are notated in a white (non-significant) to grey (increasing significance) scale.

	R132Q vs R132H		R132Q vs WT		R132H vs WT	
**GeneID**	Log2 FC	padj	Log2 FC	padj	Log2 FC	padj
AFDN	0.26	0.02	0.24	0.01	-0.02	0.98
BDNF	0.53	0.01	0.81	0.00	0.28	0.54
BMP5	1.57	0.01	0.59	0.50	-0.99	0.47
BMP7	2.01	0.01	1.03	0.34	-0.98	0.73
CBL	0.32	0.05	0.39	0.00	0.07	0.94
CDK6	0.18	0.47	0.30	0.08	0.12	0.77
COL4A5	0.61	0.01	1.16	0.00	0.55	0.00
COL4A6	0.82	0.00	1.62	0.00	0.80	0.00
EGFR	0.56	0.00	0.62	0.00	0.05	0.95
EPHB2	0.80	0.00	1.51	0.00	0.71	0.00
ETS1	0.35	0.02	0.40	0.00	0.05	0.95
FZD1	-0.47	0.00	-0.61	0.00	-0.14	0.75
ITGA2	0.99	0.00	1.49	0.00	0.50	0.00
ITGA8	2.78	0.02	1.09	0.76	NA	NA
ITGAV	0.49	0.00	0.61	0.00	0.12	0.69
ITGB1	0.27	0.05	0.48	0.00	0.22	0.21
IL6	3.24	0.05	259.00	0.04	0.07	0.99
JAG1	0.40	0.04	0.78	0.00	0.38	0.07
JAK1	0.22	0.01	0.26	0.01	0.03	0.97
JUN	0.37	0.03	0.60	0.00	0.23	0.40
KIRREL3	0.74	0.02	1.29	0.00	0.55	0.20
MET	0.30	0.10	0.48	0.00	0.17	0.60
MDM2	0.35	0.03	0.19	0.35	-0.16	0.53
NRAS	0.19	0.12	0.26	0.01	0.06	0.90
RASAL2	0.27	0.07	0.37	0.00	0.09	0.88
ROCK2	0.32	0.00	0.23	0.03	-0.09	0.83
SEMA3A	0.66	0.00	0.76	0.00	0.09	0.85
STAT3	0.23	0.08	0.44	0.00	0.20	0.12
TGFA	0.64	0.47	1.36	0.01	0.72	0.16
TGFB1	0.05	0.89	0.36	0.00	0.31	0.07
TP53	-0.22	0.42	-0.37	0.03	-0.15	0.69
TP53I3	-0.16	0.67	-0.55	0.00	-0.40	0.11
WNT10B	-1.26	0.00	-1.70	0.00	-0.44	0.67

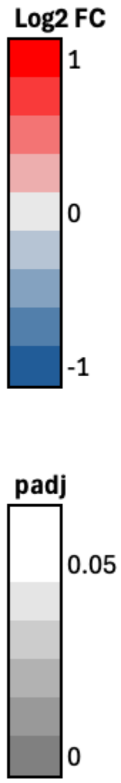

Supplement: Supporting information [file mmc1.pdf]
